# Altered Glucosinolate Profiles and Expression of Glucosinolate Biosynthesis Genes in Ringspot-Resistant and Susceptible Cabbage Lines

**DOI:** 10.3390/ijms19092833

**Published:** 2018-09-19

**Authors:** Md. Abuyusuf, Arif Hasan Khan Robin, Hoy-Taek Kim, Md. Rafiqul Islam, Jong-In Park, Ill-Sup Nou

**Affiliations:** 1 Department of Horticulture, Sunchon National University, 255 Jungang-ro, Suncheon, Jeonnam 57922, Korea; yusuf_agr@pstu.ac.bd (M.A.); gpb21bau@gmail.com (A.H.K.R.); htkim@sunchon.ac.kr (H.-T.K.); rafiq@pstu.ac.bd (M.R.I.); 2 Department of Agronomy, Patuakhali Science and Technology University, Patuakhali 8602, Bangladesh; 3 Department of Genetics and Plant Breeding, Bangladesh Agricultural University, Mymensingh 2202, Bangladesh; 4 University—Industry Cooperation Foundation, Sunchon National University, 255 Jungang-ro, Suncheon, Jeonnam 57922, Korea; 5 Department of Biotechnology, Patuakhali Science and Technology University, Patuakhali 8602, Bangladesh

**Keywords:** Ringspot, *Mycosphaerella brassicicola*, glucosinolates, R line, S line, cabbage

## Abstract

Ringspot, caused by the fungus *Mycosphaerella brassicicola*, is a serious disease of *Brassica* crops worldwide. Despite noteworthy progress to reveal the role of glucosinolates in pathogen defense, the host–pathogen interaction between cabbage (*Brassica oleracea*) and *M. brassicicola* has not been fully explored. Here, we investigated the glucosinolate profiles and expression of glucosinolate biosynthesis genes in the ringspot-resistant (R) and susceptible (S) lines of cabbage after infection with *M. brassicicola*. The concomitant rise of aliphatic glucoiberverin (GIV) and indolic glucobrassicin (GBS) and methoxyglucobrassicin (MGBS) was linked with ringspot resistance in cabbage. Pearson’s correlation and principle component analysis showed a significant positive association between GIV contents and the expression of the glucosinolate biosynthesis gene *ST5b-Bol026202* and between GBS contents and the expression of the glucosinolate biosynthesis gene *MYB34-Bol017062*. Our results confirmed that *M. brassicicola* infection induces the expression of glucosinolate biosynthesis genes in cabbage, which alters the content of individual glucosinolates. This link between the expression of glucosinolate biosynthesis genes and the accumulation of their respective glucosinolates with the resistance to ringspot extends our molecular sense of glucosinolate-negotiated defense against *M. brassicicola* in cabbage.

## 1. Introduction

Ringspot is a common fungal disease of cabbage (*Brassica oleracea*) that causes economic losses worldwide [1,2]. Ringspot is caused by the homothallic ascomycete *Mycosphaerella brassicicola*, and it is most common in countries with temperate and humid climates [3]. High humidity (>90%), temperature (5–20 °C), and moist leaf surfaces for 3–4 days are important factors for *M. brassicicola* infection [4]. Ringspot can quickly become an epidemic in cabbage, emphasizing the need for resistant varieties [5,6]. To date, only a few studies have been conducted to explore the interactions between the host and pathogen in the *B. oleracea*–*M. brassicicola* patho-system. Resistance genes and secondary metabolites involved in plant–pathogen interactions provide general resistance to pathogens and insects [7,8,9,10]. Glucosinolates (GSLs) are vital secondary metabolites found in *Brassicaceae* that are biosynthesized from amino acids, and they play anti-oxidative and anti-carcinogenic roles in humans. GSLs also play a role in resistance to insect pests and pathogens in plants. Aliphatic and indolic GSLs are the two most important types of GSLs that are present in *Brassicaceae* [11,12,13]. GSLs and their hydrolyzed products showed significant antimicrobial and insecticidal activities [14], as well as anti-fungal properties in plants [15,16,17,18,19,20,21,22]. GSLs are the precursors of sulfur- and nitrogen-containing secondary metabolites, such as iso-thiocyanates and sulforaphane [23,24,25,26]. The effects of GSL metabolism, and sulfur and nitrogen nutrition have been studied because brassica crops contain large amounts of sulfur-containing amino acids and GSLs [27,28,29,30]. In a study on the antimicrobial effect of crude extracts from *Arabidopsis thaliana* [19], 4-methylsulfonyl butyl iso-thiocyanate was found to be the main active compound, and it showed broad antimicrobial activity, thereby demonstrating the possible protective role of GSL-derived isothiocyanate against pathogens. A survey of the levels of GSLs in different brassica varieties showed alterations to the GSL profile at the time of inoculation by fungal pathogens. These changes were mainly due to an increase in aliphatic, indolic, and aromatic GSLs. In general, GSL degradation products induce the plant defense response against pathogens and general herbivores (Rask et al., 2000; Barth and Jander, 2006). Biotic and abiotic factors, such as pathogen infection, herbivore damage, mechanical injury, or mineral nutrition, modulate the GSL profile [31,32,33]. A wide range of defense reactions can affect GSL content [18,33,34,35]. However, an association between GSL levels and resistance to various fungal pathogens in brassicas has not yet been established [36].

Pathogen resistance has not been strongly correlated with the contents of GSLs following fungal infections in different brassica species [37,38]. The best in vivo evidence for the protective role of GSLs comes from the MAMl mutation study, where a decrease in GSL levels in Arabidopsis led to susceptibility to *Fusarium oxysporum* [19]. GSL levels were positively correlated with oilseed rape resistance to pathogens *Sclerotinia sclerotiorum* [39,40], with a few exceptions [34,41,42,43,44]. The inconsistency in these data may reflect individual fungal behavior (e.g., necrotrophs versus biotrophs) [45], their host specificity (e.g., brassica-specialist versus broad-spectrum) [46], the genetic purity of the host plants (e.g., isogenic versus heterozygous lines), and the amount of GSL and its degradation products that are produced by the host plant. The relationship between GSL content and its resistance to *M. braassicicola* has not yet been studied. Several recent reports have shown that resistance to obligate biotrophs, hemibiotrophs, and necrotrophs might be linked with the production of indolic GSLs in *Brassicaceae* [47,48]. These findings on the association of resistance with the GSL profile of brassica species prompted us to examine plant–pathogen interactions at the molecular and biochemical levels. Upregulation of GSL biosynthesis genes was linked with increased contents of individual GSL compounds in *B. oleracea* inbred lines [36,49,50,51]. Here, we investigated the correlation between GSL profiles in resistant and susceptible cabbage lines, and the expression of GSL biosynthesis genes upon infection with the *M. brassicicola* IPO-99008 isolate.

## 2. Results

### 2.1. Resistance of the Cabbage Lines to M. brassicicola

Inoculation of cabbage leaves with *M. brassicicola* isolate IPO-99008 resulted in different responses in the 26 cabbage lines based on the scoring criteria of ringspot disease symptoms (Table 1, Figures S1 and S3). 

**Figure 1:**
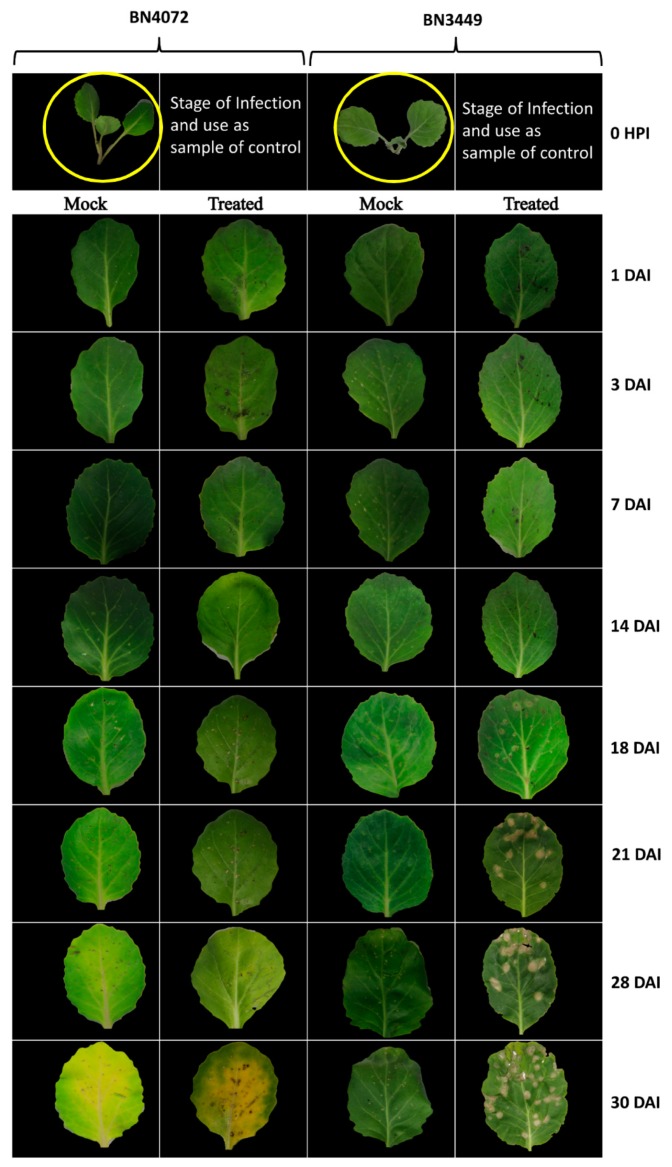
Ringspot disease progress in R line BN4072 and S line BN3449 of cabbage. Plants were infected at the three-leaf stage (yellow circles). Infected leaves were examined from 1–30 DAI (days after inoculation). HPI (Hours post inoculation), R: Resistant; S: Susceptible.

Two lines, BN4071 and BN4072 (the R line), exhibited complete resistance with no disease symptoms, while the other lines were moderately to highly susceptible to the *M. brassicicola* isolate IPO-99008 (Table 1). Disease progression was also determined in the R line BN4072, and the S line, BN3449, up to 30 DAI (days after inoculation) (Figure 1).

### 2.2. Overview of the Profile of Individual GSLs in the R and S Lines of Cabbage after M. Brassicicola Inoculation

**Figure 2:**
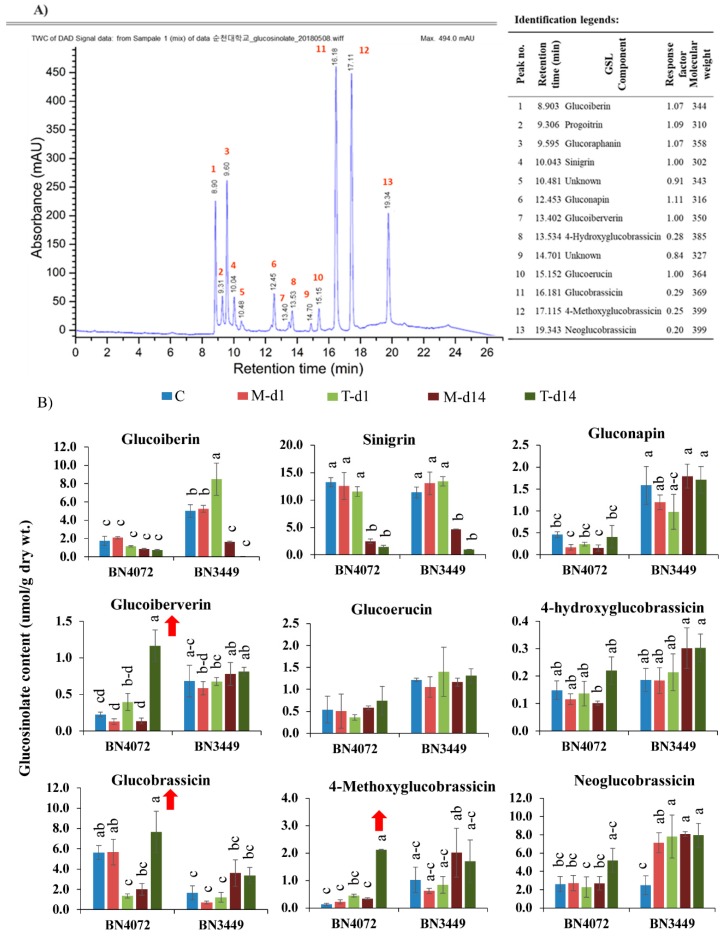
(**A**) A sample illustrative spectrum obtained from mass spectrometry analysis (HPLC/MS, Agilent 1200 series, Agilent Technologies) to identify individual GSLs of cabbage leaf extracts. Tentative determination of peaks, Retention time (min), Response factors were performed as indicated in the identification legends. (**B**) Individual glucosinolate contents of leaf samples from ringspot R line BN4072 and S line BN3449 of cabbage under different treatments (C: Control; M-d1: Mock day 1; T-d1: Treated day 1; M-d14: Mock day 14; T-d14: Treated day 14). The means of three biological replicates are shown. Vertical bars indicate standard deviation. Different letters indicate statistically significant differences between R and S lines, and treatment interactions. Upward-pointing red arrows indicate increased glucosinolate content in response to *Mycosphaerella brassicicola* infection. R: Resistant; S: Susceptible.

Individual glucosinolate compounds identified in cabbage leaves of different samples are shown in Figure 2A and Supplementary file 1 and 2. HPLC analysis identified nine types of GSL compounds (glucoiberin (GIB), sinigrin (SIN), glucoerucin (GER), gluconapin (GNA), glucoiberverin (GIV), hydroxyglucobrassicin (HGBS), glucobrassicin (GBS), mythoxyglucobrassicin (MGBS), and neoglucobrassicin (NGBS)) in the R line, BN4072, and the S line, BN3449, of cabbage. In uninfected control plants, the amount of both aliphatic and indolic GSLs (i.e., GIB, GNA, and GBS) varied significantly between the R and S lines (Figure 2B). In the control plants, GIB and GNA levels were higher in the S line compared to the R line, whereas the opposite was observed for GBS. Inoculation of third-leaf stage cabbage plants with *M. brassicicola* fungal isolate IPO-99008 significantly changed the GSL profiles in the leaves of the R and S lines. In the R line, the levels of GIV significantly increased by 8.58-fold at 14 DAI compared to the mock-treated plants (Figure 2B). In contrast, the levels of GIV were not significantly altered after fungal infection in the S line (Figure 2B, Table S5). The levels of MGBS significantly increased by 6.20-fold at 14 DAI in the R line compared to the mock-treated plants, whereas the levels of MGBS were not significantly altered in the S line after infection. (Figure 2B, Table S5). The levels of GBS increased by 3.82-fold in the R line compared to the mock-treated plants at 14 DAI (Figure 2B, Table S5), whereas there were no significant changes in GBS levels in the S line after infection compared to the mock-treated plants (Figure 2B). 

GIV and MGBS showed an increasing trend only in the R line after infection. In the S line, GIB was significantly increased by 1.62-fold at 1 DAI compared to the mock-treated plants (Figure 2B). Overall, our results showed that the contents of both aliphatic GIV and indolic GBS and MGBS increased in the R line at the time of infection (Figure 2B). By contrast, the level of GIB was increased in the S line after infection. In addition, total GSLs were increased in the R line, but they were decreased in the S line at 14 DAI (Figure S6).

### 2.3. Upregulation of MYB34-Bol017062, MYB34-Bol036262, ST5b-Bol026202, and ST5c-Bol030757 in the R Line after Inoculation

**Figure 3:**
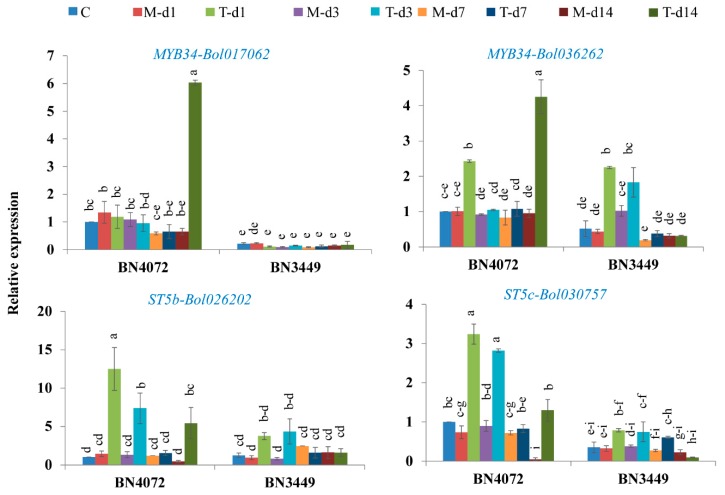
Remarkable upregulation of *MYB34-Bol017062*, *MYB34-Bol036262*, *ST5b-Bol026202*, and *ST5c-Bol030757* genes in Ringspot R line BN4072 at 14 DAI compared to mock-treated plants. C: Control; M-d1: Mock day 1; T-d1: Treated day 1; M-d3: Mock day 3; T-d3: Treated day 3; M-d7: Mock day 7; T-d7: Treated day 7; M-d14: Mock day 14; T-d14: Treated day 14. The means of three biological replicates are shown. Vertical bars indicate standard deviation. Different letters indicate statistically significant differences between R line BN4072 and S line BN3449, and treatment interactions. R: Resistant; S: Susceptible.

The expression levels of 38 GSL pathway-related genes are given in the Supplementary file 1 and 3. Among the aliphatic and indolic GSL biosynthesis genes, only one indolic GSL transcription factor-related gene, *MYB34-Bol017062*, showed a 9.25-fold upregulation at 14 DAI in the R line (Figure 3, Table 2, and Table S2). The following genes were also significantly upregulated in the R line at 14 DAI compared to the mock-treated plants: *MYB34-Bol036262* by 4.46-fold, *ST5b-Bol026202* by 14.07-fold, and *ST5c-Bol030757* by 27.31-fold (Figure 3). 

### 2.4. Upregulation of Transcription Factor-Related Genes and GSL Biosynthesis Genes in the S Line after Inoculation

**Figure 4:**
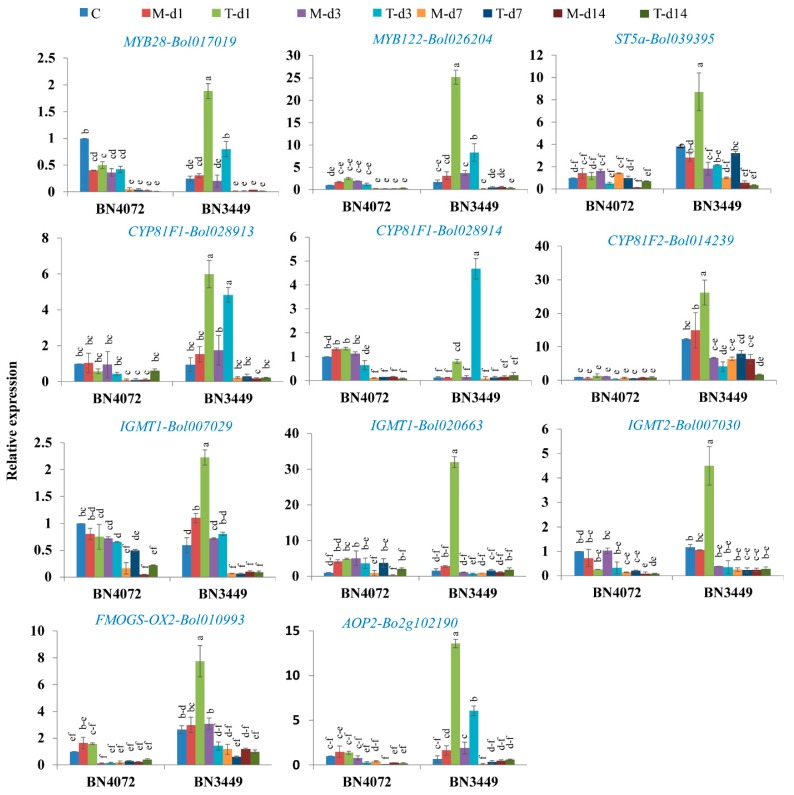
Upregulated genes of transcription factor related and glucosinolate biosynthesis genes in Ringspot S line BN3449 of cabbage. C: Control; M-d1: Mock day 1; T-d1: Treated day 1; M-d3: Mock day 3; T-d3: Treated day 3; M-d7: Mock day 7; T-d7: Treated day 7; M-d14: Mock day 14; T-d14: Treated day14. The means of three biological replicates are shown. Vertical bars indicate standard deviation. Different letters indicate statistically significant differences between R and S lines, and treatment interactions. R: Resistant; S: Susceptible.

The expression levels of 11 GSL biosynthesis genes were measured in the control, mock-treated, and infected plants. In the uninfected control plants, one transcription factor, *MYB28-Bol017019*, involved in aliphatic GSL biosynthesis, and one indolic GSL biosynthesis gene, *IGMT1-Bol007029*, showed significantly higher expression in the R line compared to the S line (Figure 4). In the S line at 1 DAI, *MYB28-Bol017019* and *MYB122-Bol026204*, which encode transcription factor-related genes related to the aliphatic and indolic GSLs, had increased expression (6.1- and 8.0-fold, respectively) compared to the mock-treated plants (Figure 4, Table 2 and Table S2). In addition, the aliphatic GSL biosynthesis genes *FMOGS-OX2-Bol010993* and *AOP2-Bo2g102190* had 2.6- and 8.3-fold higher expression, respectively, in the S line at 1 DAI, compared to the mock-treated plants (Figure 4, Table 2 and Table S3). 

The expression levels of seven indolic GSL biosynthesis genes were also increased in the S line after inoculation with *M. braassicicola* compared to the mock-treated plants: *ST5a -Bol039395* by 3.1-fold, *CYP81F1-Bol028913* by 3.9-fold, *CYP81F2-Bol014239* by 1.8-fold, *IGMT1-Bol007029* by 2.0-fold, *IGMT1-Bol020663* by 11.4-fold, and *IGMT2-Bol007030* by 4.3-fold at 1 DAI, and *CYP81F4-Bol028918* by 5.6-fold at 3 DAI (Figure 4, Table 2 and Table S4).

### 2.5. Upregulation of Transcription Factor-Related Genes and GSL Biosynthesis Genes in the R and S Lines

Among the 38 transcription factor-related genes and GSL biosynthesis genes, the expression of 12 genes were higher in the S line compared to the R line after inoculation (Table 2, Figure S4). *MYB28-Bol036743*, *MYB29-Bol008849*, and *MYB51-Bol030761*, which encode transcription factors related to the aliphatic and indolic GSLs, had 4.0-, 11.0-, and 4.9-fold increased expression, respectively, in the S line at 1 DAI, compared to the mock-treated plants (Table 2 and Table S2, Figure S4). In the R line, increased expression was found in the indolic GSL biosynthesis pathway genes compared to the mock-treated plants as follows: *CYP81F4-Bol032712* by 15.44-fold at 1 DAI, *CYP81F3-Bol032711* by 9.26-fold at 3 DAI, *CYP81F2-Bol026044* by 12.86-fold at 1 DAI and 16.08-fold at 14 DAI, and *CYP81F4-Bol032714* by 15.35-fold at 1 DAI (Figure S4, Table 2 and Tables S2–S4). Conversely, the upregulation of these genes in the S line after infection compared to the mock-treated plants was lower than that observed in the R line: *CYP81F4-Bol032712* by 6.1-fold at 1 DAI, *CYP81F3-Bol032711* by 5.69-fold at 3 DAI, *CYP81F2-Bol026044* by 3.3-fold at 1 DAI and 1.25-fold at 14 DAI, and *CYP81F4-Bol032714* by 22.8-fold (5-fold lower than in the R line) at 1 DAI (Figure S4, Table 2 and Tables S2–S4). In the control plants, the expression levels of one aliphatic transcription factor-related gene, *MYB28-Bol036286*, and two indolic GSL biosynthesis genes, *CYP81F1-Bol017375* and *CYP81F1-Bol017376*, were higher in the R line than in the S line and some asymmetrical response of expression were found in both the R and S lines (Figure S5). 

### 2.6. Correlation between the Levels of Individual GSLs and the Expression Level of GSL Biosynthesis Pathway Genes Induced by M. brassicicola in the R and S Lines 

**Figure 5:**
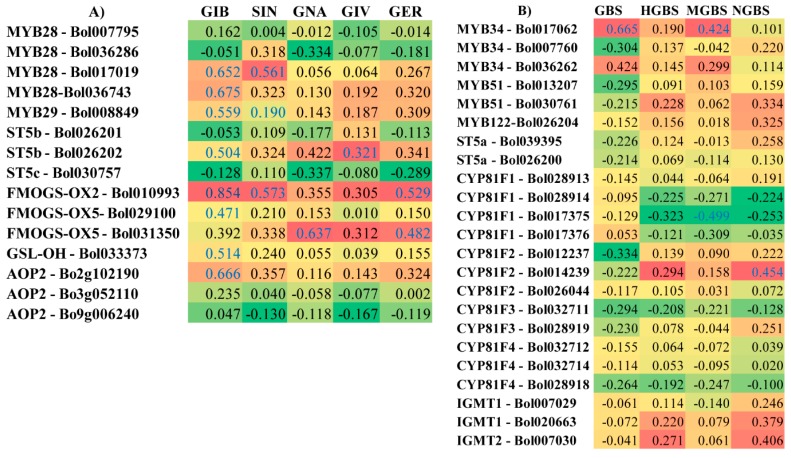
Heat maps showing correlation between the levels of aliphatic (**A**) and indolic (**B**) glucosinolate component and expression of biosynthesis genes under four specific treatments (M-d1: Mock day 1; T-d1: Treated day 1; M-d14: Mock day 14; T-d14: Treated day 14) in Ringspot R line BN4072 and S line BN3449 of cabbage. Blue letters represent statistically significant correlation (*p* < 0.05). For each gene and glucosinolate combination, the values indicate Pearson correlation coefficient. Red cells represent positive correlation, and green cells represent negative correlation. GIB: Glucoiberin; SIN: sinigrin; GNA: gluconapin, GIV: glucoiberverin; GER: glucoerucin; GBS: glucobrassicin; NGBS: neoglucobrassicin; MGBS: methoxyglucobrassicin; and HGBS: hydroxyglucobrassicin. R: Resistant; S: Susceptible.

A heat map emphasized that the fold changes in the expression levels of transcription factor-related genes and GSL biosynthesis genes observed after pathogen inoculation were consistent with the changes in the levels of individual GSLs measured in the R and S lines, compared to their mock-treated samples. Pearson’s correlation coefficients showed the highest significant positive correlation with the aliphatic GSLs GIB, SIN, and GER with *FMOGS-OX2-Bol010993* expression; whereas a positive correlation was also observed for SIN with *MYB28-Bol017019* expression; GNA with *FMOGS-OX5-Bol031350* expression; and GIV with *ST5b-Bol026202* expression (Figure 5A). In the case of indolic GSLs, GBS, and MGBS had the highest significant positive correlation with *MYB34-Bol017062* expression but NGBS had the highest significant positive correlation with *CYP81F2-Bol014239* expression (Figure 5B). 

**Figure 6:**
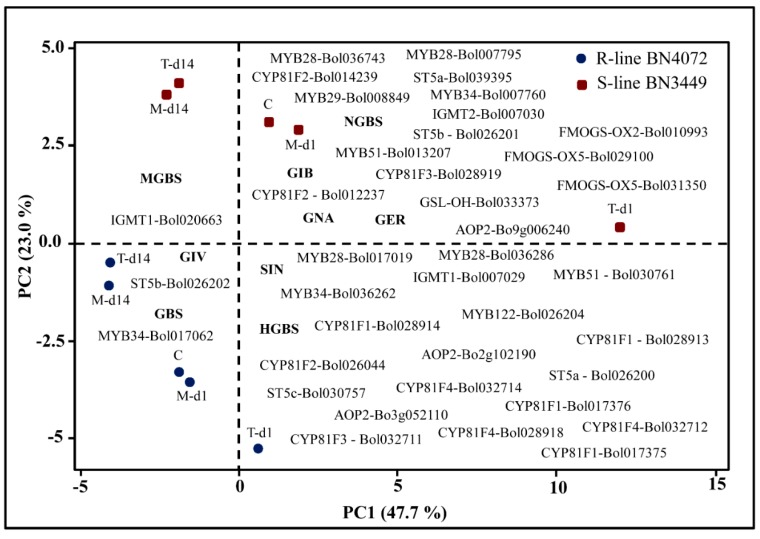
Biplot of cabbage ringspot R and S lines, glucosinolate components, and responses of glucosinolate pathway genes (denoted within figure) as determined by principle component analysis (PCA). Red squares denote the mean PC scores of the R line BN4072, and blue circles denote the mean PC scores of S line BN3449. *Mycosphaerella brassicicola* treatments indicated by C: Control; M-d1: Mock day 1; T-d1: Treated day 1; M-d14: Mock day 14; T-d14: Treated day14. Glucosinolate (GSL) components are GIB: glucoiberin; SIN: sinigrin; GNA: gluconapin, GIV: glucoiberverin; GER: glucoerucin; GBS: glucobrassicin; NGBS: neoglucobrassicin; MGBS: methoxyglucobrassicin; and HGBS: hydroxyglucobrassicin. R: Resistant; S: Susceptible.

These data show the association of these genes with changes in the GSL profile. Principal component analysis (PCA) of the contents of the nine individual GSL components and the expression of the GSL biosynthesis pathway genes revealed the accumulation of individual GSL components and gene expression towards ringspot R line BN4072 and S line BN3449 of cabbage. There was a major contrast among the gene expression patterns. The first four PCs explained 90.2% of the total variation in the datasets (Table S8). In PC1, which accounted for 47.7% of the total variation, was largely demonstrated by higher positive coefficients for NGBS, GIB, GNA, GER, SIN, and HGBS versus lower negative coefficients for GIV, GBS, and MGBS. The other positive and negative coefficients for gene expression are presented in Supplementary Table S8. However, PC2 explained 23.0% of the total variation, which largely showed a contrast between the positive coefficients for MGBS, NGBS, GIB, GNA, and GER and the negative coefficients for GIV, SIN, GBS, and HGBS. PC2 clearly distinguished the R line from the S line based on GSL profiles, and the expression of the GSL biosynthesis genes (Figure 6 and Table S8). 

## 3. Discussion

### 3.1. Levels of Total GSLs, GIV, and GBS Are Related to Ringspot Resistance

We found a link between pathogen-induced GSL accumulation and plant resistance. There was a correlation with the GSL concentration and the resistance level in cabbage. Increased total GSLs in the R line and decreased total GLSs in the S line were correlated to the resistance or susceptibility in cabbage, respectively (Table S5). These findings were supported by the results that showed that reduced GSL levels in Arabidopsis caused susceptibility to *F. oxysporum* [19], and that a wide spectrum of defense reactions can affect GSL content [18,33,34,35]. In previous studies, pathogenic resistance in different brassica species was not strongly correlated with the overall level of GSLs [37,38]. In *Brassica napus*, a negative correlation was reported between *Alternaria* infection and GSL levels [41,42], and a positive correlation was reported between pathogen-induced accumulation of indolic GSLs and *Sclerotinia sclerotiorum* infection [52]. In our study, the levels of individual GSLs were often altered in the mock-treated plants compared to the control plants. For example, the contents of GNA, GIV, GER, GBS, HGBS, and NGBS were generally flexible in the mock-treated compared to control plants in the R line and S line (Figure 2B We evaluated the changes of the individual GSLs of the mock-treated samples as a reference. The concentration of aliphatic GIV and indolic GBS and MGBS GSLs increased in the R line after inoculation (Figure 2B), indicating that ringspot resistance in cabbage may be accomplished through an accumulation of both aliphatic and indolic GSLs. These results were consistent with some studies [36], but different from other studies [45,48,53,54]. These data suggest that increased levels of aliphatic GIV, and indolic GBS and MGBS may confer resistance to *M. brassicicola* in cabbage.

### 3.2. Increased Expression of ST5b-Bol026202 Led to Increased Levels of GIV in the R Line

Secondary alterations of the desulfoglucosinolates GIB and GIV, and other aliphatic GSLs are linked with the *ST5b* and *ST5c* genes (Figure S2). In this study, increased expression of *ST5b-Bol026202* was associated with higher levels of GIV biosynthesis (Figure 5A), which was supported by a previous study [49]. Therefore, our results indicate that infection-induced upregulation of these genes lead to an increase in the levels of GIV, which was linked with ringspot resistance (Figure 2B and Figure 3), but associations based on these correlations should be further validated through molecular studies.

### 3.3. Increased Levels of Aliphatic GIV and Indolic GBS and MGBS Are Associated with Ringspot Resistance

The increased level of GIV observed in the R line and a somewhat reduced static level of GIV observed in the S line suggested that GIV plays an important role in GSL-mediated resistance (Figure 2B). In addition, the increased levels of indolic GBS and MGBS observed in the R line compared to the S line also likely contribute to ringspot resistance in cabbage (Figure 2B). These results were consistent with the higher accumulation of GIV, GBS, and NGBS associated with blackleg resistance in cabbage [51]. 

### 3.4. Expression of MYB34 Likely Induces the Expression of GSL Biosynthesis Genes, Leading to Increases in GBS and MGBS in the R Line

*MYB34* genes directly control the biosynthesis of indolic GSLs in Arabidopsis [55] and *B. oleracea* [49,50]. *MYB34* together with *MYB51* and *MYB122* impart resistance to *Plectosphaerella cucumerina*, where PENETRATION2 (*PEN2)* played a vital role in triggering the expression of indolic GSL biosynthesis genes in response to pathogen infection [53]. *MYB34* (Bol007760) is also induced in broccoli (*Brassica oleracea* var. *italica*) when treated with methyl jasmonate (MeJA), which was associated with jasmonic acid (JA) signaling [50]. In this study, the expression of *MYB34-Bol017062* and *MYB34-Bol036262* increased by 9.25- and 4.46-fold, respectively, at 14 DAI with *M. brassicicola* in the R line compared to the mock-treated plants (Figure 3). *MYB34-Bol017062* and *MYB34-Bol036262* may play a role in the trans-activation of genes required for the biosynthesis of indolic GSLs in response to *M. brassicicola* infection (Figure 2B). In our results, the upregulation of the expression of *MYB34-Bol017062* and *MYB34-Bol036262* in the R line was associated with the accumulation of indolic GBS and MGBS which was supported by the results [49]. On the contrary, a number of genes, including *MYB28-Bol017019*, *MYB122-Bol026204*, *AOP2-Bo2g102190*, *FMOGS-OX2-Bol010993*, *ST5a-Bol039395*, *CYP81F1-Bol028913*, *CYP81F1-Bol028914*, *CYP81F2-Bol014239*, *CYP81F3-Bol028919*, *CYP81F4-Bol028918*, *IGMT1-Bol007029*, *IGMT1-Bol020663*, and *IGMT2-Bol007030*, were highly expressed only in the S line after inoculation (Table 2, Figure 4). However, these changes in expression did not contribute to significant changes in the accumulation of the linked indolic and aliphatic GSLs in the R and S lines. Therefore, this observation requires further investigation. In a recent study, a particular line displayed relatively low levels of total GSLs, indicating that the expression of certain biosynthesis genes is not always consistent with higher accumulation of GSLs from the relevant biosynthesis pathways [49].

### 3.5. Accumulation of Indolic GBS and MGBS in the R Line is Activated by Increased Expression of GSL Biosynthesis Genes

The concentrations of GBS and MGBS, which play a role in antifungal responses in plants, increased in the R line in response to *M. brassicicola* infection, which was associated with a significant upregulation of the expression of *CYP81F4-Bol032712*, *CYP81F3-Bol032711*, *CYP81F2-Bol026044*, and *CYP81F4-Bol032714* (Figure S4). In a previous study, a MeJA treatment increased the expression of *CYP81F4* by 2,400-fold in broccoli and 10-fold in cabbage [50], suggesting that resistance is governed by signaling pathways involved in the metabolism of indolic GSLs. A previous study [56] also stated a similar association between *CYP81F2* expression and GBS levels in *B. oleracea*. The accumulation of GBS and MGBS and the upregulation of *CYP81F1*, *CYP81F2*, *CYP81F3*, *CYP81F4*, *IGMT1*, and *IGMT2* (Figure 4 and Figure S4) in cabbage raises the question of whether the accumulation of endogenous GSLs always reflects a physiological response at any given time. The quantity of GSLs in leaf tissues is a result of concomitant activation of myrosinases (biosynthesis and catabolism), which can upregulate the contents of the GSL components at a specific time period. MGBS levels increased by 30–47% in response to *Leptospharia maculans* after 5–8 days of infection in *B. napus* [57]. In vitro studies have reported the antifungal activity of MGBS, as well as SIN and GBS [58] and increased accumulation of MGBS conferred moderate resistance in cabbage plants to *L. maculans*. [36]. Here, we observed an increase in the levels of MGBS in the R line compared to the S line after *M. brassicicola* infection (Figure 2B) along with upregulation of *CYP81F4-Bol032712*, *CYP81F3-Bol032711*, *CYP81F2-Bol026044*, and *CYP81F4-Bol032714*, which are involved in methoxylation and the conversion of GBS to 4-MGBS. It is likely that both GSL contents and GSL biosynthesis pathway genes serve to confer resistance to *M. brassicicola*. Our findings also agree with the report that *CYP81F2* induces antifungal defenses [47]. 

### 3.6. Accumulation of Aliphatic GIV with Expression of ST5b-Bol026202 and Indolic GBS with MYB34-Bol017062 May Play a Role in Resistance 

Our heat map showed a consistent relationship between gene expression and the contents of individual GSLs in the R and S lines. Pearson’s correlation coefficient showed the highest significant positive correlation in the levels of the aliphatic GSL GIV with the expression of *ST5b-Bol026202* and with the levels of the indolic GSL GBS with the expression of *MYB34-Bol017062* (Figure 5). These data show that changes in the status of these genes correlate to the contents of individual GSLs in response to *M. brassicicola* infection. This result is consistent with a recent study for higher expression of *MYB34* genes under *L. maculans* inoculation [36] and methyl jasmonate elicitation [50]. PCA showed a major difference between the accumulation of individual GSL components and gene expression in the R and S lines. In the PCA biplot, GIV is closely linked with *ST5b-Bol026202* and GBS is closely linked with *MYB34-Bol017062*, and they are located in one of the four quadrants **(**Figure 6). These GSLs might function in the regulation of resistance to ringspot in cabbage. These results were also supported by a previous observation that a simultaneous increase in the contents of aliphatic and indolic GSLs, and the expression of their associated genes (i.e., GIV and GER with *ST5b-Bol026201*, *ST5b-Bol026202*, and *GSL-OH-Bol033373*, GBS with *MYB34-Bol007760* and *ST5a-Bol026200*, and NGBS with *CYP81F4-Bol032712*, *Bol032714*, and *CYP81F2-Bol026044*) were correlated with complete resistance to blackleg in cabbage [36]. Lastly, the upregulation of GSL biosynthesis genes occurred within one to three days after inoculation, whereas the symptoms appeared later. GSLs began to accumulate 14 days later when disease symptoms started to appear. This suggests that prior to visible symptoms, GSL biosynthesis genes were induced in order to initiate the GSL-mediated resistance response.

### 3.7. Association of GSL Biosynthesis Genes and Accumulation of Individual GSLs in the S Line

Eleven GSL biosynthesis genes were highly expressed in the S line at one to three DAI (Figure 4). The PCA analysis showed some association among individual GSLs and the S line (i.e., GIB, GNA, GER, NGBS, and MGBS) (Figure 6). Among these individual GSLs, only GIB significantly accumulated in the S line (Figure 2B). Therefore, aliphatic GIB may confer susceptibility to *M. brassicicola* in cabbage. This result was also supported by the observation that the highest levels of aliphatic GIB were present in a cabbage line highly susceptible to blackleg [36].

## 4. Materials and Methods

### 4.1. Plant Materials and Growth Conditions

Seeds of 26 cabbage inbred lines (Table 1) (*B. oleracea* var. *capitata*) were germinated in multi-pot trays using coco-peat soil in a growth chamber at 24 °C, 60% relative humidity (RH), and a 16/8 h (light/dark) photoperiod. When the first two leaves became visible, the plants were transferred to larger pots (10 × 10 × 12 cm) filled with a mixture of 50% coco-peat and 50% soil. Plants were inoculated at the third-leaf stage and were grown inside the growth chamber (17 °C, 98% RH) for one month after inoculation.

### 4.2. Inoculum Preparation

*M. brassicicola* isolate IPO-99008 was collected from the Stichting Dienst Landbouwkundig Onderzoek (DLO), Research institute Praktijkonderzoek Plant & Omgeving/Plant Research International, Wageningen, The Netherlands and grown on V8 agar at 17 °C under a 12 h ultraviolet (380 nm) light/12 h dark photoperiod. Four weeks before inoculation, small pieces of mycelium (1–2 mm) were transferred to fresh V8 agar to initiate new colonies. Prior to inoculation, eight of these colonies were suspended in 250 mL of distilled water using a micro-blender. The number of spores in the suspensions that could act as infectious units was determined by counting individual hyphae fragments (7.6 × 10^5^ infection units per mL) in a hemocytometer [4]. 

### 4.3. Inoculation Technique 

Twenty-six inbred lines with five replicates from each line were inoculated at the third-leaf stage by spraying approximately 3 mL of mycelial suspension per plant with a T-1440A plastic hand pump sprayer (www.apolloind.co.kr, Apollo ind. Co. ltd, Sihung-si, Korea). Approximately 5–7 wounds per cm^2^ leaf area were created on the leaves by a needle. The inoculated plants were covered with plastic bags to maintain a high humidity. After six days, the plastic bags were removed and the inoculated plants were stored in a growth chamber (17 °C, 98% RH). Symptoms were scored from 1 to 30 days after inoculation (DAI). In addition, five replicates of a ringspot resistant line, BN4072, (R line) and a susceptible line, BN3449, (S line), were used as a control (not wounded or inoculated) and another five replicates were mock-treated (wounded but not infected). 

### 4.4. Disease Assessment

A 5-point grading scale based on the percentage of diseased leaf surface was used to rate the progression of disease development (Figure S1). The grading scale was as follows: 1 = 0%, 2 = 1–5%, 3 = 6–20%, 4 = 21–50%, and 5 = greater than 50% infected leaf surface. Plant lines rated 1–2 were considered resistant (R), those rated 3 were considered moderately resistant (MR), lines rated 4 were considered moderately susceptible (MS), and those rated 5 were considered very susceptible (VS) [59]. 

### 4.5. Leaf Sampling and Preparation for High-Performance Liquid Chromatography and Gene Expression Analysis

Leaf samples were taken from three randomly selected plants of the R line and the S line from each of the control, mock-treated, and *M. brassicicola* infected plants in September 2017 to evaluate the levels of endogenous GSLs, and to quantify the expression of GSL biosynthetic pathway genes (Figure S2). Leaf samples collected for HPLC analysis and reverse transcription quantitative PCR (RT-qPCR) were flash-frozen in liquid nitrogen and immediately stored at −80 °C.

### 4.6. Assessment of GSL Content

Leaf samples from three biological replicates from each of the control, mock-treated, and *M. brassicicola* infected plants were used to extract desulfoglucosinolates via a modified HPLC protocol as previously described [49,50,60]. Frozen leaf tissue stored at −80 °C were treated with methanol and pulverized to a fine powder. Powdered leaf samples were stored at 70 °C for 10 min and then kept at room temperature for about one hour. The powdered samples were centrifuged at 10,000× *g* for 8 min at 4 °C to remove undesirable sediments. The supernatant collected at the end of the anion-exchange chromatography was considered as the crude GSL sample. The crude GSL was then desulfurized as previously described [49,50,60]. The desulfurized GSLs were eluted with 1 mL of distilled water. The eluted desulfoglucosinolates were purified with high-speed centrifugation at 20,000× *g* for 4 min at 4 °C followed by filtering through a polytetrafluoroethylene filter (13 mm, 0.2 μm, Advantec, Pleasanton, CA, USA). Purified GSLs were analyzed by HPLC on a Waters 2695 HPLC system (Waters, Milford, MA, USA) equipped with a C18 column (Zorbax Eclipse XBD C18, 4.6 × 150 mm, Agilent Technologies, Palo Alto, CA, USA). Water and acetonitrile were used as the mobile phase solvents. Individual GSL contents were measured using a PDA996 UV-visible detector (Waters) at a wavelength of 229 nm. Sinigrin (SIN) was used to prepare the standard curve for quantification of the identified GSLs. Mass spectrometry analysis (HPLC/MS, Agilent 1200 series, Agilent Technologies) was used to identify individual GSLs [50].

### 4.7. Primer Design for Expression Analysis of GSL Biosynthesis Genes

We selected 38 genes involved in GSL biosynthesis pathways, of which 11 genes encode transcription factors: five from the aliphatic and six from the indolic biosynthesis pathways. Of the remaining 27 genes, 10 are aliphatic GSL biosynthesis genes, and 17 are indolic GSL biosynthesis genes (Table S1, Figure S2) [49,50]. Primers were previously designed and primer efficiency was tested [49].

### 4.8. cDNA Synthesis and RT-qPCR Analysis

Total RNA was extracted from the collected leaf samples using the RNeasy mini kit (Catalog No. 74106, Qiagen, Valencia, CA, USA). cDNA synthesis was performed from total RNA using a PrimeScript-based kit (Takara Bio, Inc., Shiga, Japan). The iTaqTM SYBRR Green Super-mix was used with ROX (Bio-Rad, Hercules, Calif., USA) to perform quantitative PCR. For each reaction, a total volume of 20 μL was prepared containing 10 μL PCR master mix, 7 μL ultra-pure water, 2 μL forward and reverse primers, and 1 μL cDNA template with a concentration of 60 ng·μL^−1^. The PCR conditions were as follows: denaturation at 95 °C for 10 min followed by 40 cycles of denaturation at 95 °C for 20 s, annealing at 58 °C for 20 s, and amplification and signal acquisition at 72 °C for 30 s. Data were recorded as fluorescence intensity at the end of each cycle for each sample. Each biological replicate was tested in three technical replicates. Quantification (Cq) analysis was done using Light Cycler 96 software (Roche, Mannheim, Germany). The relative expression of each sample was calculated using Livak’s comparative 2^−∆∆*C*t^ method [61]. Three different actin primers, *actin1*, *actin2*, and *actin3* [49], were designed from three actin genes selected from the NCBI database. Genes (GenBankAccession Nos. AF044573 [62], JQ435879 [63], and XM_013753106 [64]) were expressed in the R and S lines, and were used as a reference. 

### 4.9. Statistical Analysis

One-way analysis of variance (ANOVA) was performed to test the statistical significance of the different treatments between the R line, BN4072, and the S line, BN3449, using Minitab 18 statistical software (Minitab Inc., State College, PA, USA). The heat map was drawn in Microsoft Excel to show the correlation between GSL content and GSL biosynthetic gene expression according to a specific treatment on the R and S lines using conditional formatting options (Tables S2–S5). Pairwise comparison of Tukey’s posthoc test was performed to visualize the statistical significance among the treatment interactions. Test statistics, degrees of freedom, and *p*-values of statistical significance for GSL content and the relative expression of GSL biosynthetic genes are shown in Tables S6 and S7. *ST5b-Bol026202* and between GBS contents and the expression of the glucosinolate biosynthesis gene *MYB34-Bol017062*

## 5. Conclusions

In this study, we screened 26 cabbage lines against *M. brassicicola* that causes ringspot disease. A completely resistant line BN4072 and a highly susceptible line BN3449 to ringspot disease were then selected to investigate gene expression and glucosinolate metabolite profiles. GSL profiling and expression analysis of GSL-related genes in cabbage infected by *M. brassicicola* identified a direct association between the expression of the genes and the contents of their corresponding GSLs in a resistant and a susceptible line of cabbage. This study showed that the simultaneous accumulation of pathogen-induced aliphatic GIV, indolic GBS, and indolic MGBS were associated with ringspot resistance in cabbage. Remarkably higher expressions of indolic transcription factor *MYB34-Bol017062*, indolic biosynthesis gene *CYP81F2-Bol026044* and aliphatic biosynthesis genes *ST5b-Bol026202* and *ST5b-030757* at 14 days after inoculation were notable. The GSLs and their corresponding genes identified in this study are candidate genetic and biochemical determinants of resistance and could be tested in efforts to improve ringspot resistance in cabbage.

## Figures and Tables

**Table 1:** Results of the disease assay of the 26 *Brassica oleracea* lines to evaluate the levels of resistance in cabbage to ringspot based on the following grading criteria: 1 = 0%, 2 = 1–5%, 3 = 6–20%, 4 = 21–50%, and 5 = greater than 50% infected leaf surface. Plant lines rated 1–2 are considered resistant (R), those rated 3 are considered moderately resistant (MR), lines rated 4 are considered moderately susceptible (MS), and those rated 5 are considered very susceptible (VS). DAI: Days after inoculation.

Sl No.	Line Name	24 DAI	30 DAI
1	BN3122	MS	MS
2	BN3270	MS	VS
3	BN3273	MS	VS
4	BN3305	MS	MS
5	BN3328	MS	MS
6	BN3383	MS	MS
7	BN3412	VS	VS
8	BN3414	MS	VS
9	BN3449	VS	VS
10	BN3631	VS	VS
11	BN3637	MS	MS
12	BN4066	MS	MS
13	BN4071	R	R
14	BN4072	R	R
15	BN4073	MS	MS
16	BN4074	MS	VS
17	BN4085	MS	VS
18	BN4098	VS	VS
19	BN4105	MS	VS
20	BN4118	MS	VS
21	BN4161	MS	VS
22	BN4168	MS	VS
23	BN4303	MS	VS
24	BN4320	MS	VS
25	BN106	MS	MS
26	BN107	MS	VS

**Table 2:** Differential expression of glucosinolate biosynthesis pathway-related genes in the ringspot-resistant (R) line, BN4072, and the susceptible (S) line, BN3449, of cabbage. Green and red color indicate days at which genes are expressed (DAI) and fold-changes compared to the mock-treated plants, respectively.

Gene Functions	Upregulated Genes in the R Line BN4072	Upregulated Genes in the S Line BN3449	Upregulated Genes in Both the R Line and S Line	
			Higher in the R Line	Higher in the S Line
Transcription factor related genes	*MYB34-Bol017062* (14–9.25)	*MYB28-Bol017019* (1–6.1) *MYB122-Bol026204* (1–8.0)	*MYB34-Bol036262* (14–4.46)	*MYB51-Bol030761* (1–4.9) *MYB28-Bol036743* (1–4.0) *MYB29-Bol008849* (1–11.0)
Aliphatic biosynthesis		*AOP2-Bo2g102190* (1–8.3) *FMOGS-OX2-Bol010993* (1–2.6)	*ST5b-Bol026202* (14–14.07) *ST5c-Bol030757* (14–27.31)	GSL-OH -Bol033373 (3–2.43) *AOP2-Bo3g052110* (3–14.05) *AOP2-Bo9g006240* (3–6.21)
Indolic biosynthesis		*ST5a-Bol039395* (1–3.1) *CYP81F1-Bol028913* (1–3.9) *CYP81F1-Bol028914* (3–30.43) *CYP81F2-Bol014239* (1–1.8) *IGMT1-Bol007029* (1–2.0) *IGMT1-Bol020663* (1–11.4) *IGMT2-Bol007030* (1–4.3)	*CYP81F4-Bol032712* (1–15.44) *CYP81F3-Bol032711* (3–9.26) *CYP81F2-Bol026044* (14–16.08) *CYP81F4-Bol032714* (1–15.35)	*ST5a-Bol026200* (1–4.5) *CYP81F3-Bol028919* (1–4.3) *CYP81F4-Bol028918* (3–5.6)

## Supplementary Materials

Supplementary materials can be found at http://www.mdpi.com/1422-0067/19/9/2833/s1. Reference [65] is cited in the supplementary materials.
