# Supplementary material for: Altered Glucosinolate Profiles and Expression of Glucosinolate Biosynthesis Genes in Ringspot-Resistant and Susceptible Cabbage Lines
Source: Int J Mol Sci. 2018 Sep 19;19(9):2833. doi: 10.3390/ijms19092833 (PMC6163659; doi:10.3390/ijms19092833)
Supplement: Supplementary file 1 [file ijms-19-02833-s001.zip › Supplementary Figures.pptx]

## Slide 1
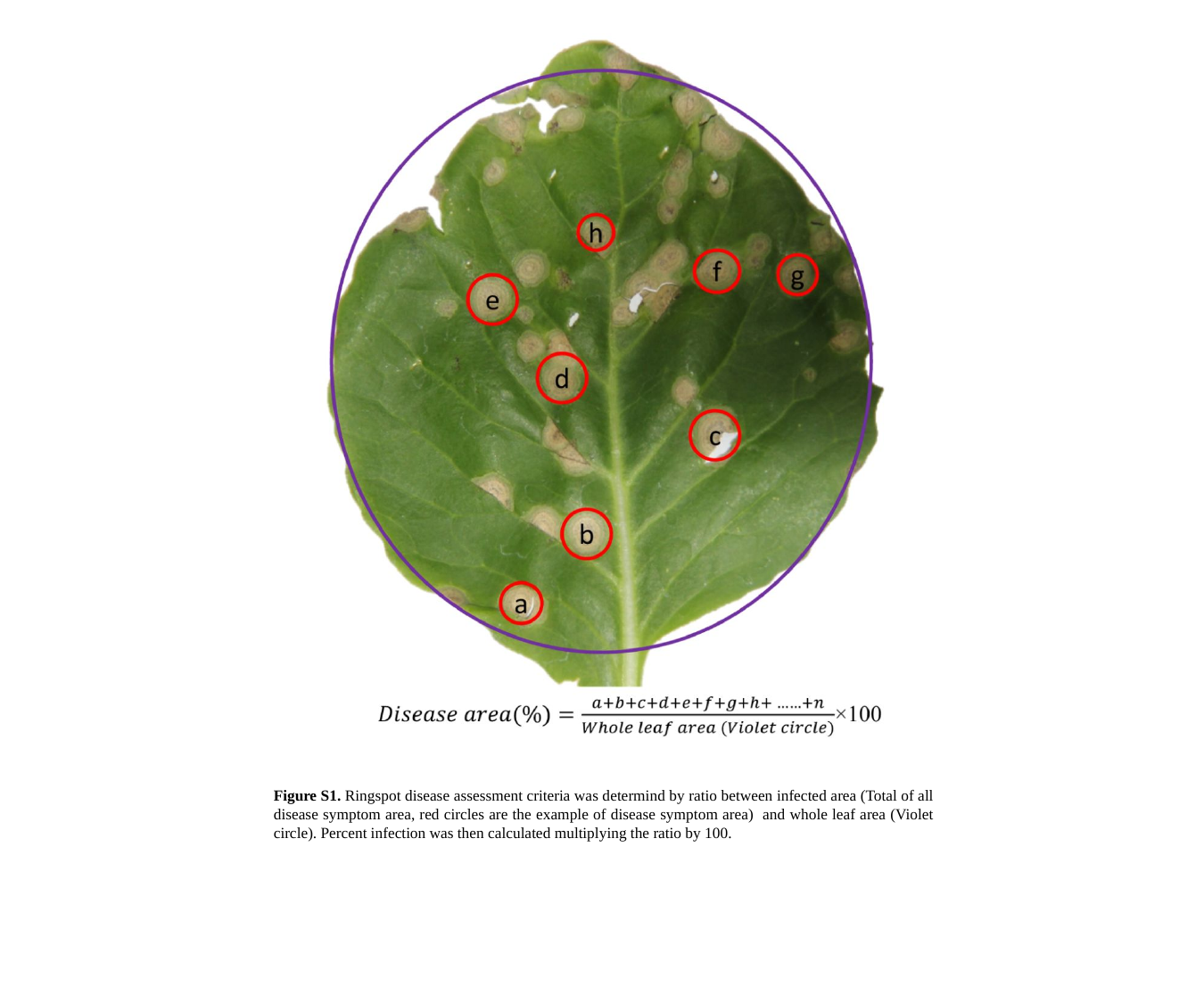

Figure S1. Ringspot disease assessment criteria was determind by ratio between infected area (Total of all disease symptom area, red circles are the example of disease symptom area) and whole leaf area (Violet circle). Percent infection was then calculated multiplying the ratio by 100.

## Slide 2
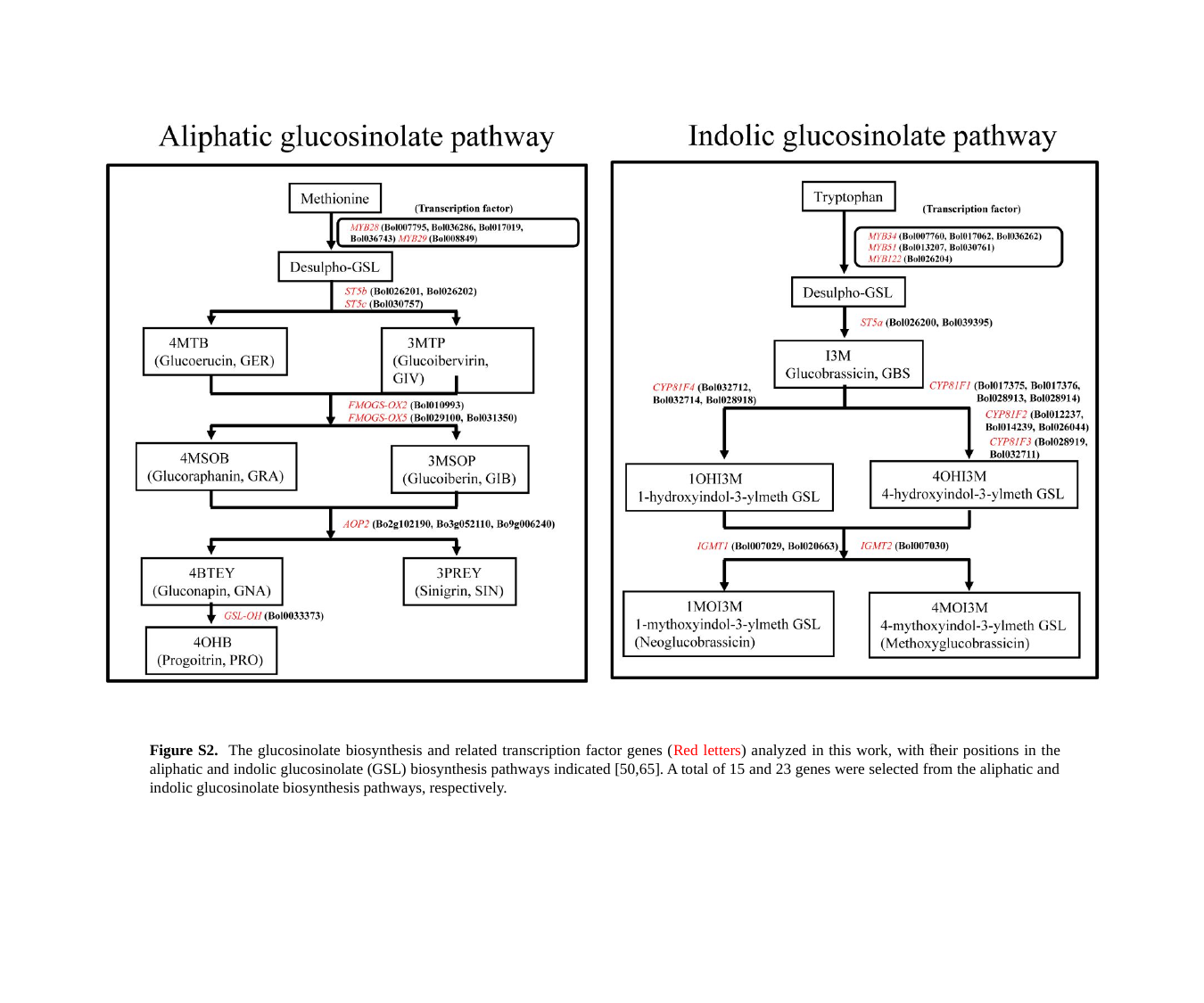

Figure S2. The glucosinolate biosynthesis and related transcription factor genes (Red letters) analyzed in this work, with their positions in the aliphatic and indolic glucosinolate (GSL) biosynthesis pathways indicated [50,65]. A total of 15 and 23 genes were selected from the aliphatic and indolic glucosinolate biosynthesis pathways, respectively.
2

## Slide 3
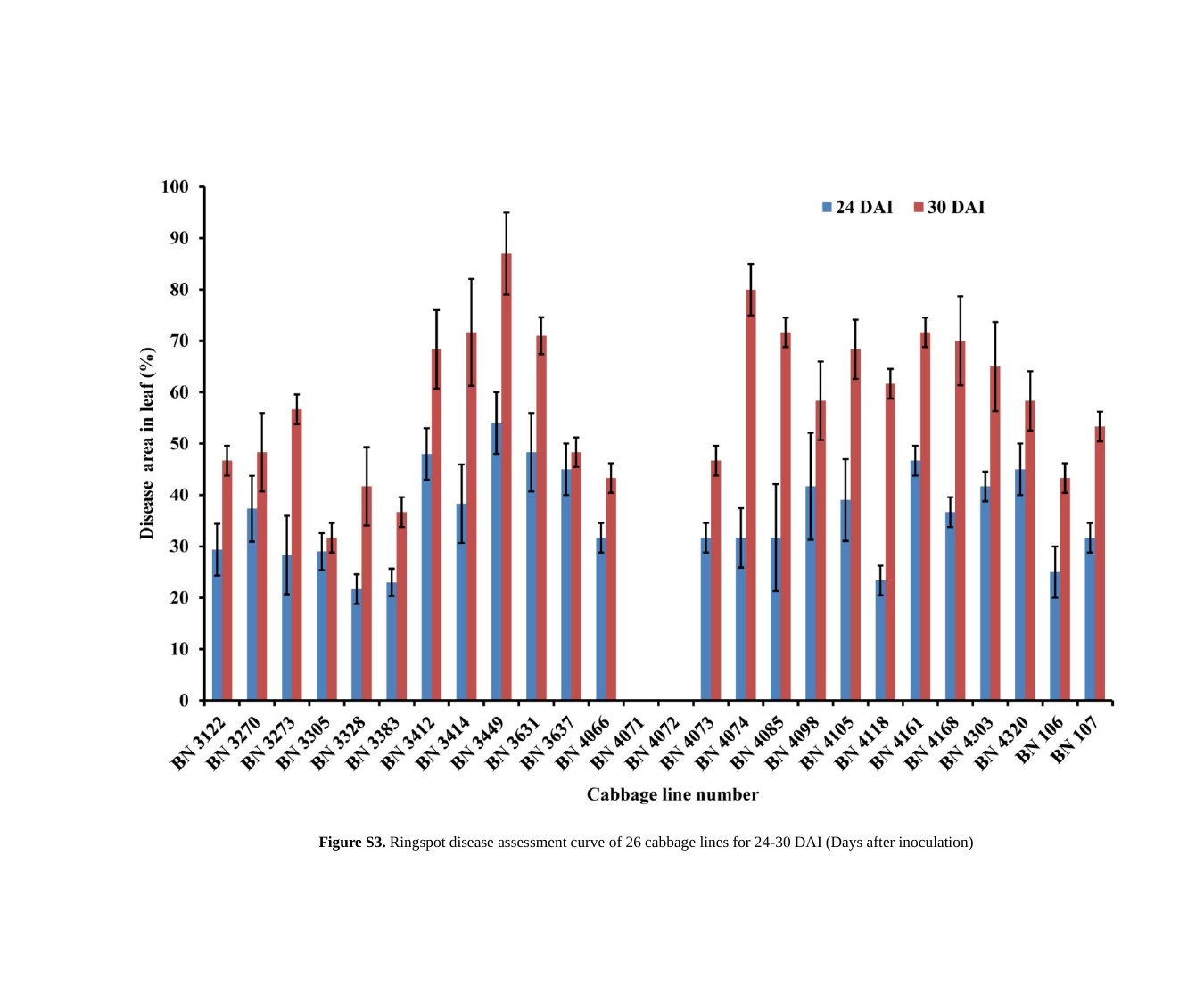

Figure S3. Ringspot disease assessment curve of 26 cabbage lines for 24-30 DAI (Days after inoculation)

## Slide 4
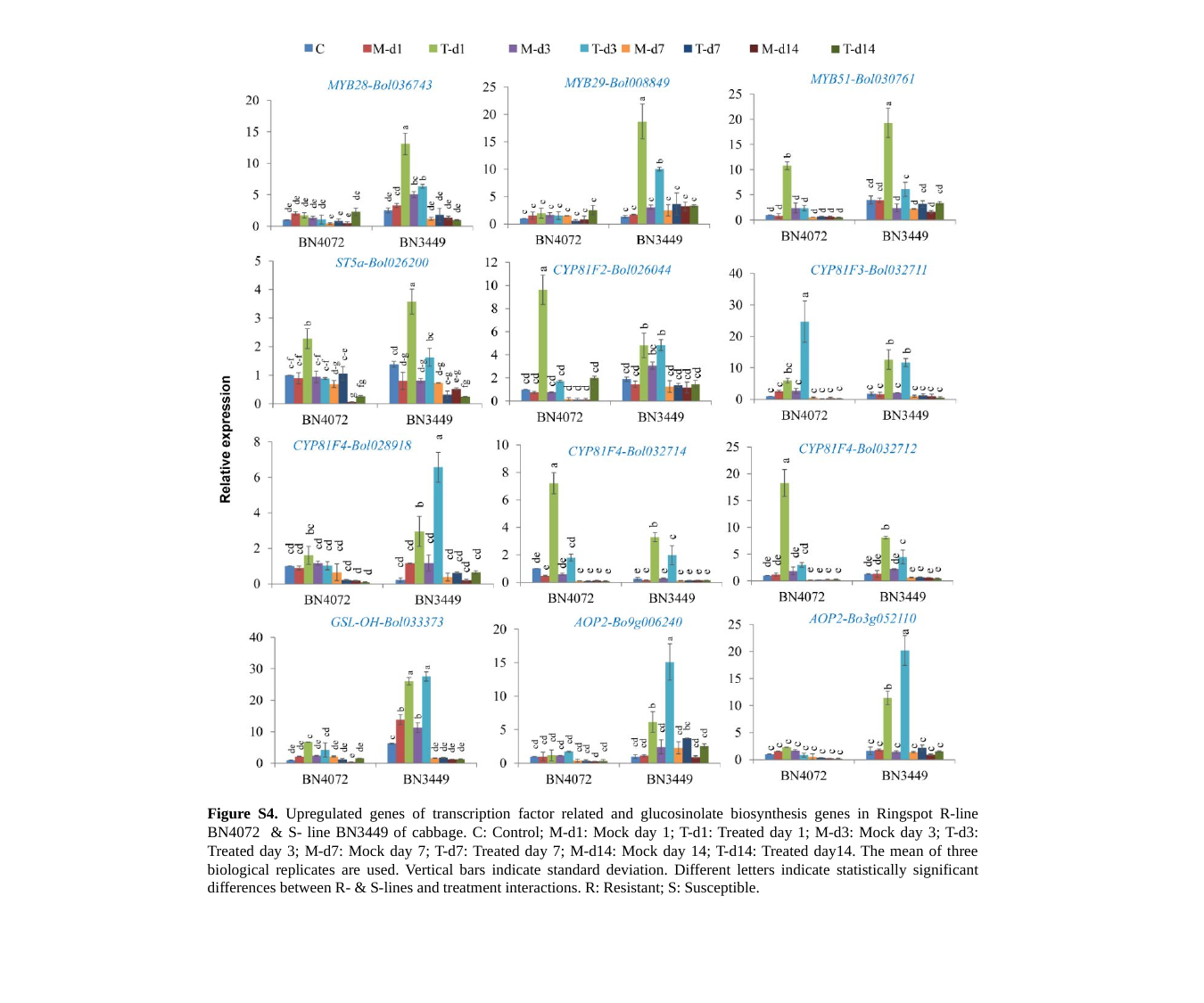

Figure S4. Upregulated genes of transcription factor related and glucosinolate biosynthesis genes in Ringspot R-line BN4072 & S- line BN3449 of cabbage. C: Control; M-d1: Mock day 1; T-d1: Treated day 1; M-d3: Mock day 3; T-d3: Treated day 3; M-d7: Mock day 7; T-d7: Treated day 7; M-d14: Mock day 14; T-d14: Treated day14. The mean of three biological replicates are used. Vertical bars indicate standard deviation. Different letters indicate statistically significant differences between R- & S-lines and treatment interactions. R: Resistant; S: Susceptible.

## Slide 5
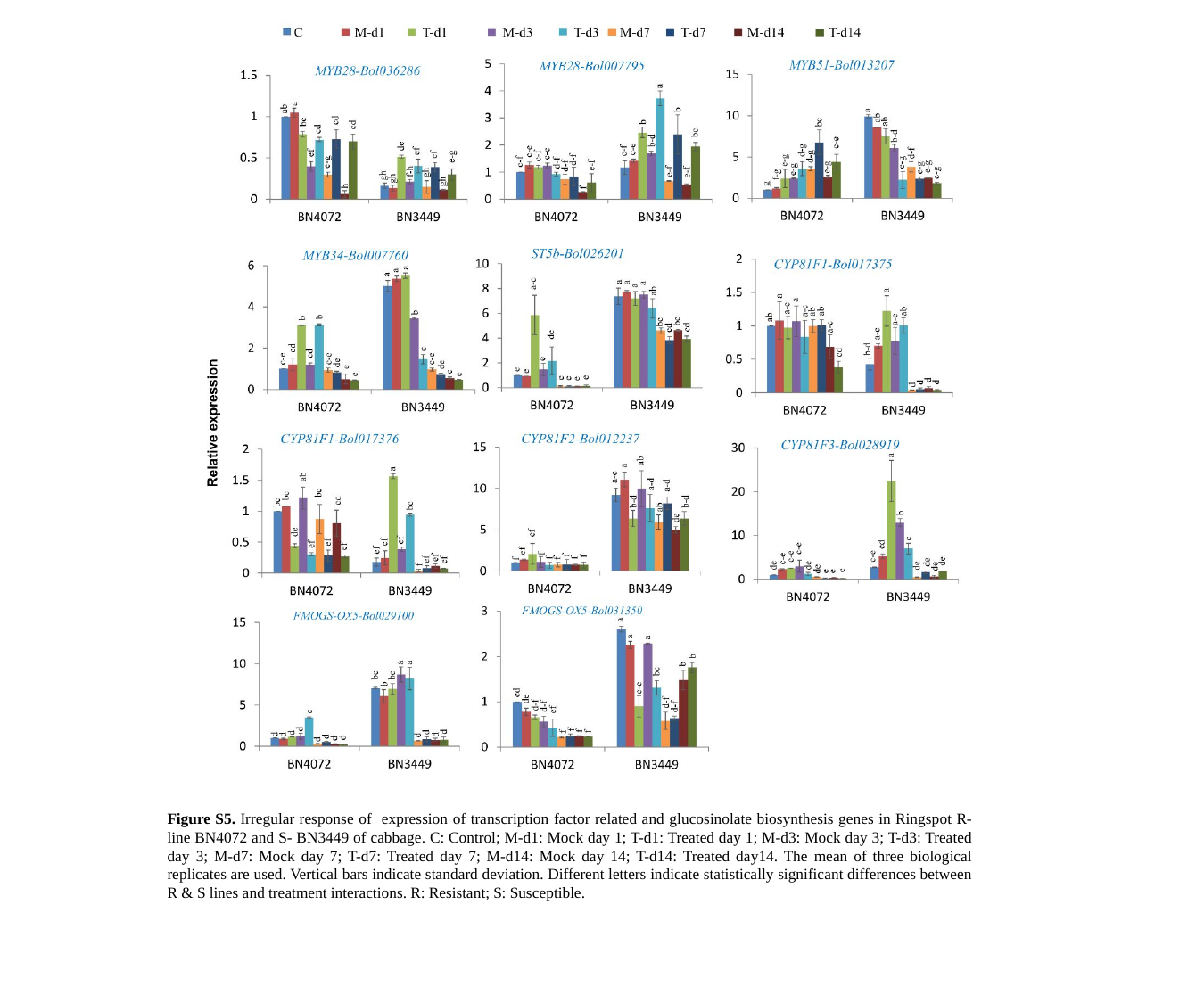

Figure S5. Irregular response of expression of transcription factor related and glucosinolate biosynthesis genes in Ringspot R-line BN4072 and S- BN3449 of cabbage. C: Control; M-d1: Mock day 1; T-d1: Treated day 1; M-d3: Mock day 3; T-d3: Treated day 3; M-d7: Mock day 7; T-d7: Treated day 7; M-d14: Mock day 14; T-d14: Treated day14. The mean of three biological replicates are used. Vertical bars indicate standard deviation. Different letters indicate statistically significant differences between R & S lines and treatment interactions. R: Resistant; S: Susceptible.

## Slide 6
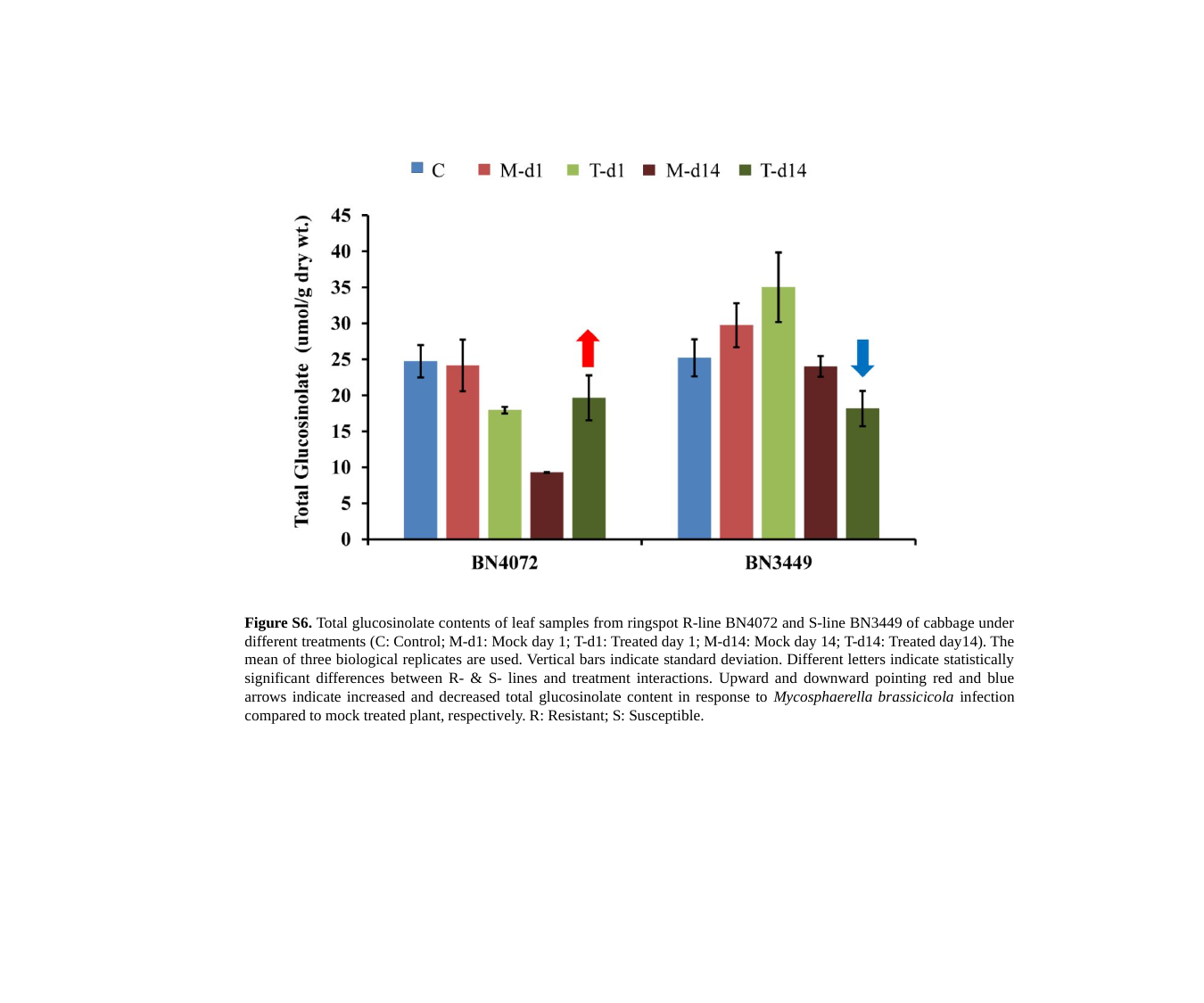

Figure S6. Total glucosinolate contents of leaf samples from ringspot R-line BN4072 and S-line BN3449 of cabbage under different treatments (C: Control; M-d1: Mock day 1; T-d1: Treated day 1; M-d14: Mock day 14; T-d14: Treated day14). The mean of three biological replicates are used. Vertical bars indicate standard deviation. Different letters indicate statistically significant differences between R- & S- lines and treatment interactions. Upward and downward pointing red and blue arrows indicate increased and decreased total glucosinolate content in response to Mycosphaerella brassicicola infection compared to mock treated plant, respectively. R: Resistant; S: Susceptible.
