# Supplementary material for: Altered Glucosinolate Profiles and Expression of Glucosinolate Biosynthesis Genes in Ringspot-Resistant and Susceptible Cabbage Lines
Source: Int J Mol Sci. 2018 Sep 19;19(9):2833. doi: 10.3390/ijms19092833 (PMC6163659; doi:10.3390/ijms19092833)
Supplement: Supplementary file 1 [file ijms-19-02833-s001.zip › Supplementary file 2.pptx]

## Slide 1
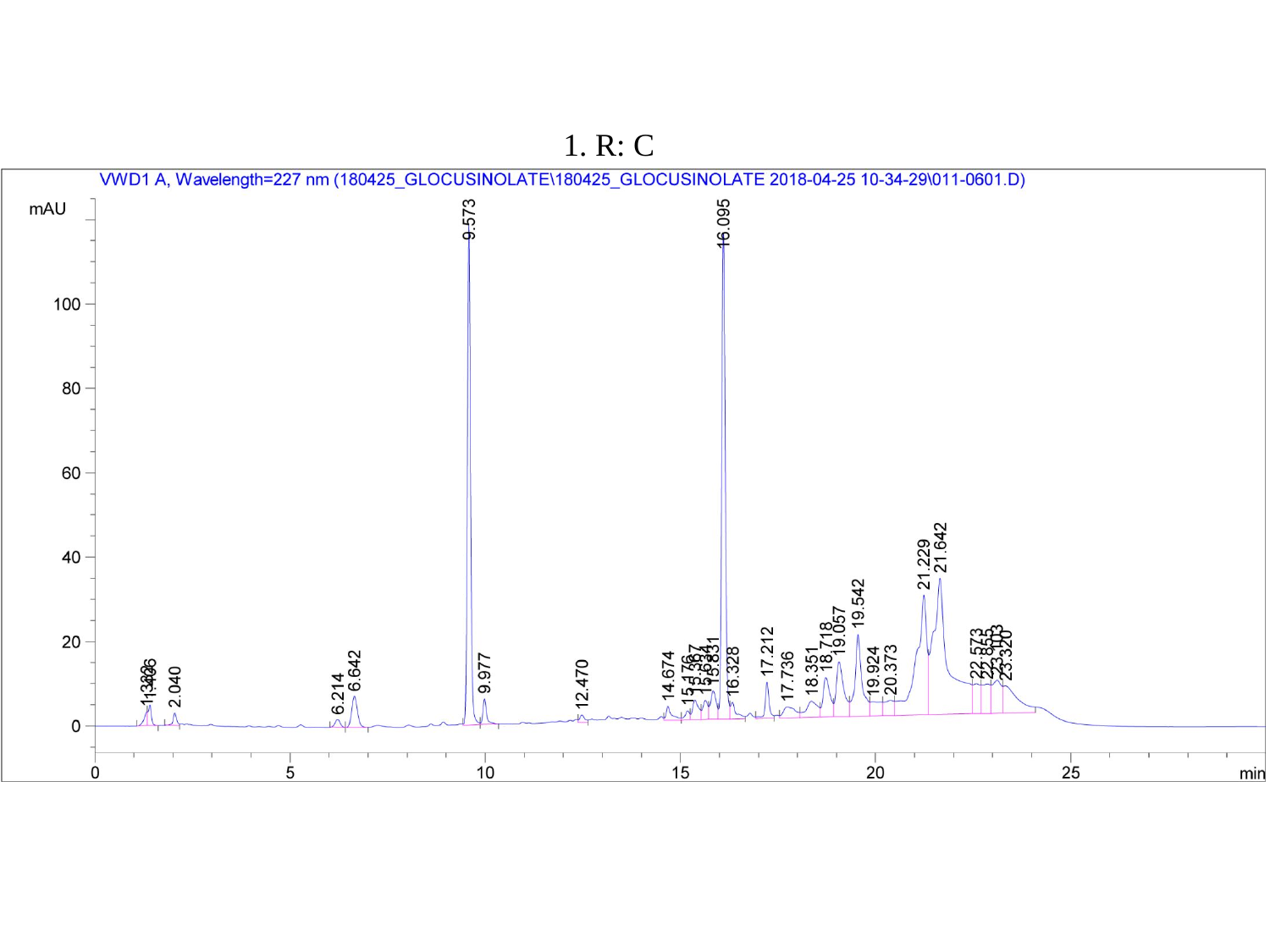

1. R: C

## Slide 2
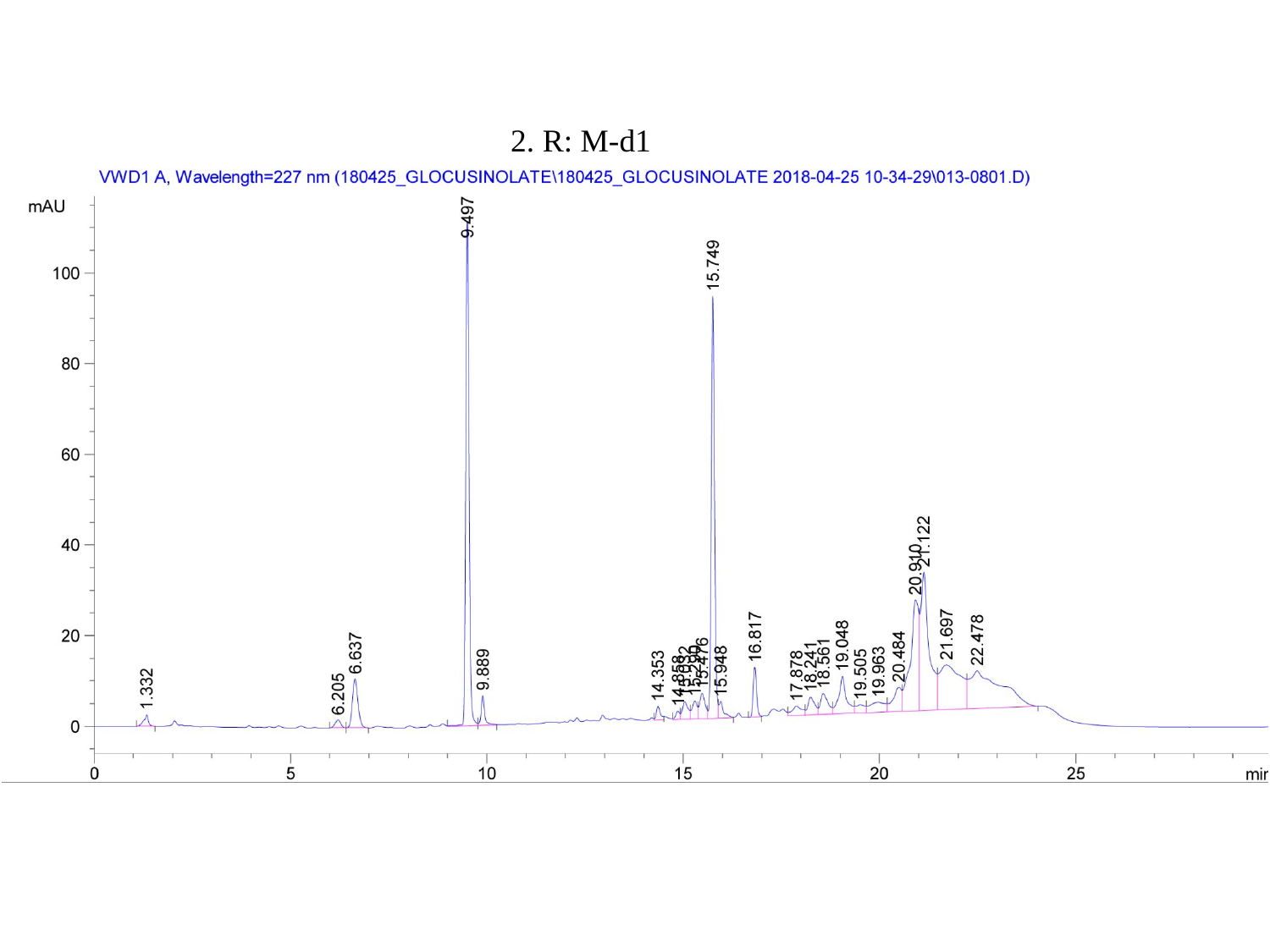

2. R: M-d1

## Slide 3
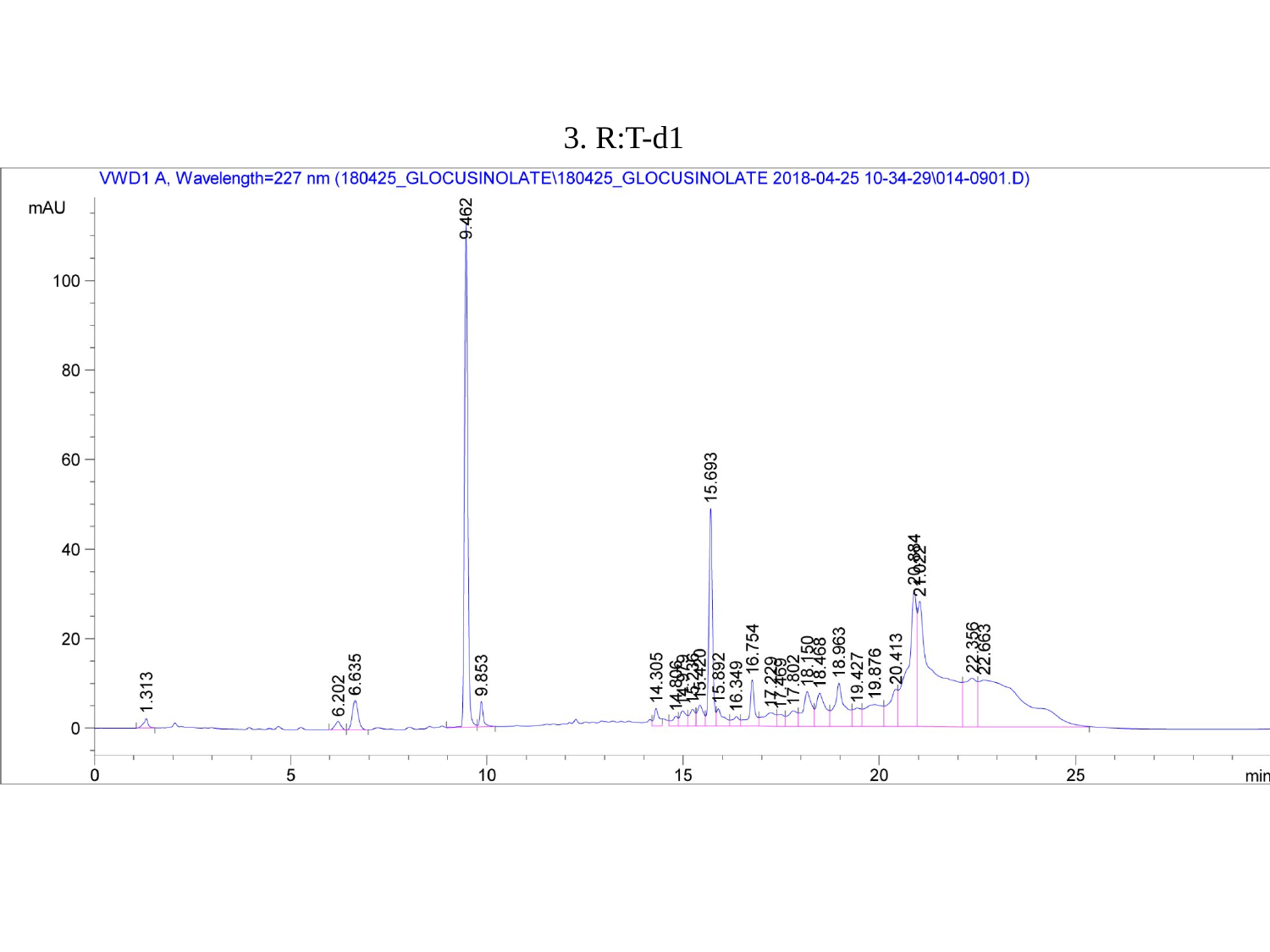

3. R:T-d1

## Slide 4
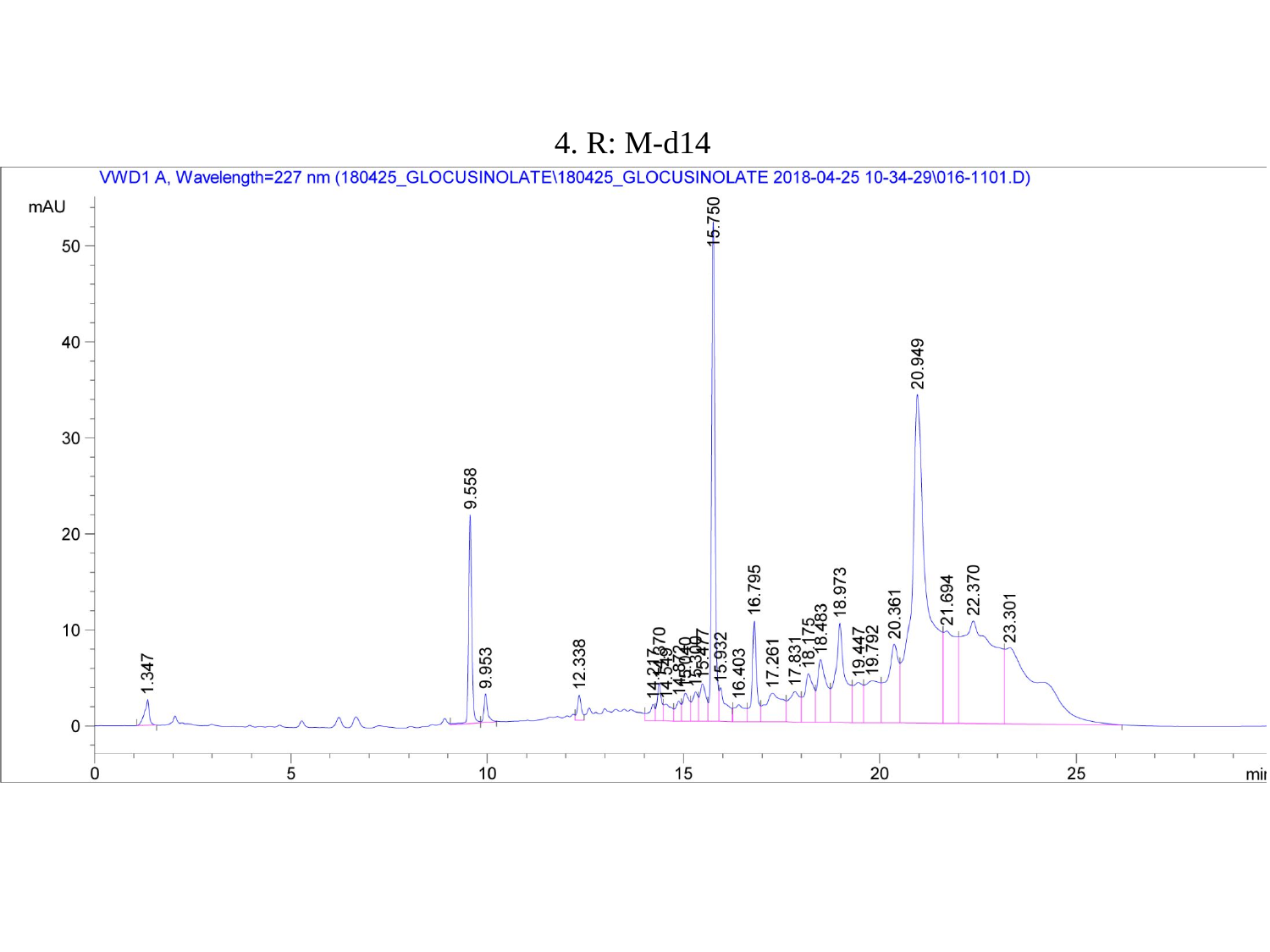

4. R: M-d14

## Slide 5
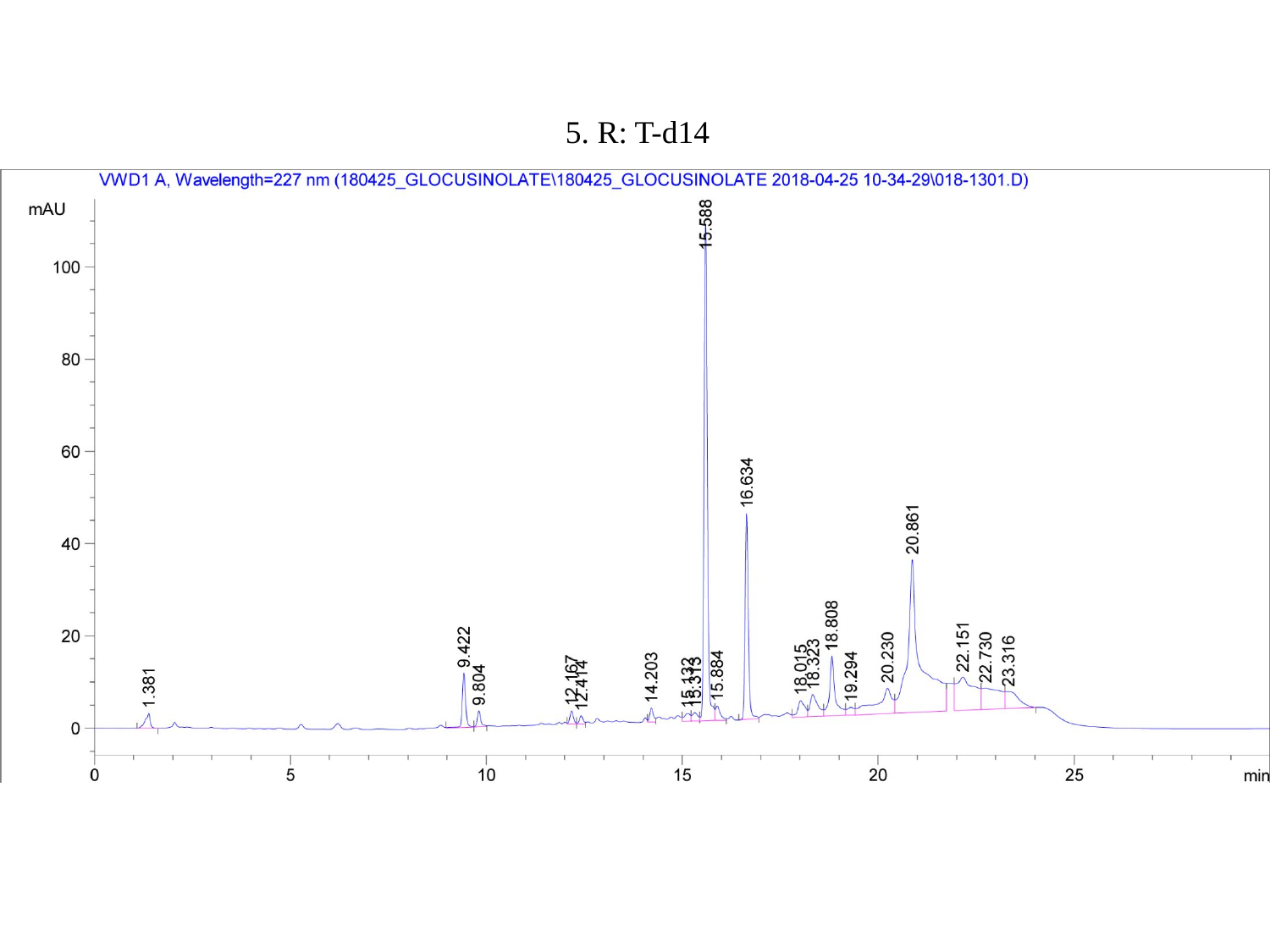

5. R: T-d14

## Slide 6
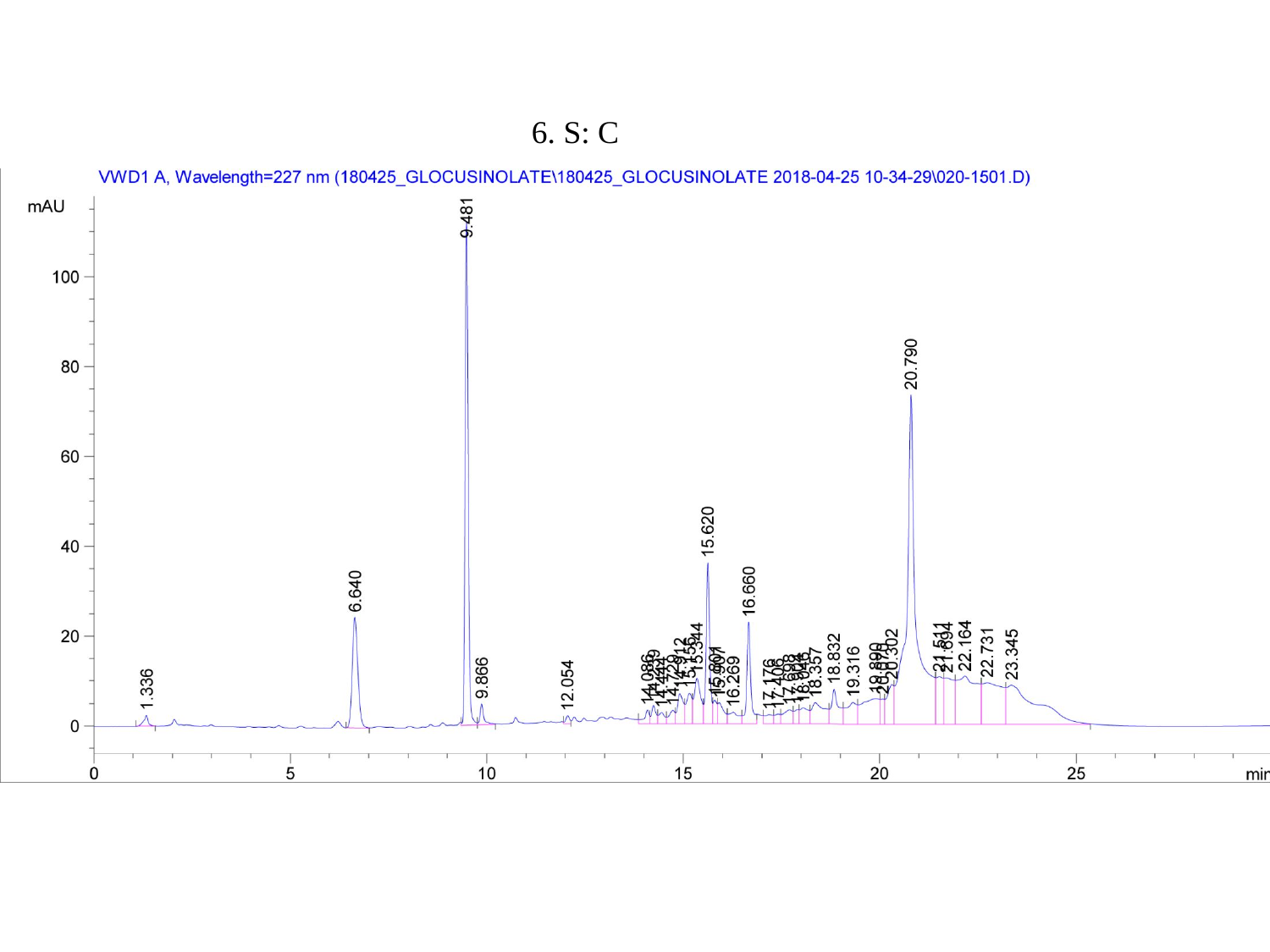

6. S: C

## Slide 7
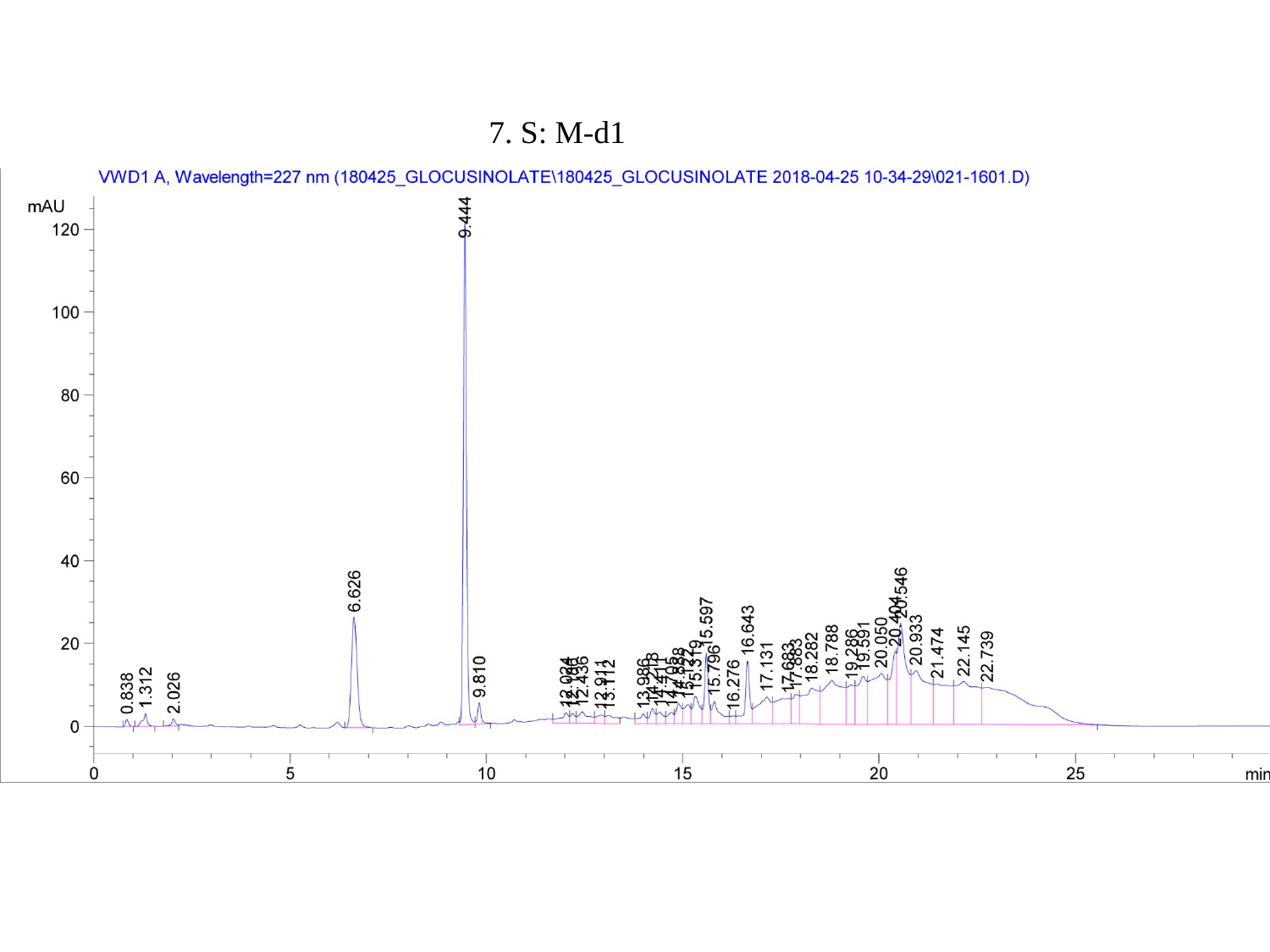

7. S: M-d1

## Slide 8
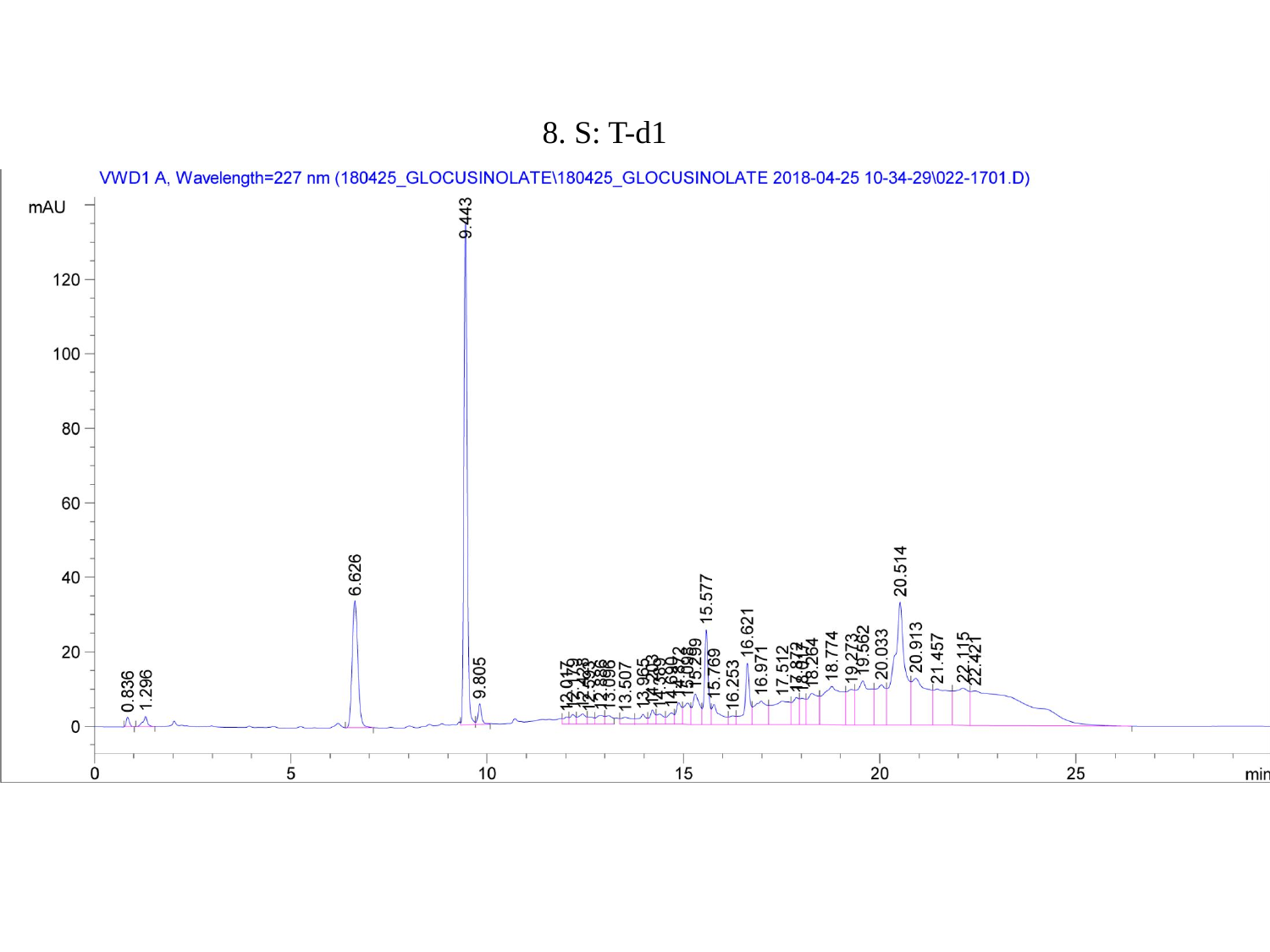

8. S: T-d1

## Slide 9
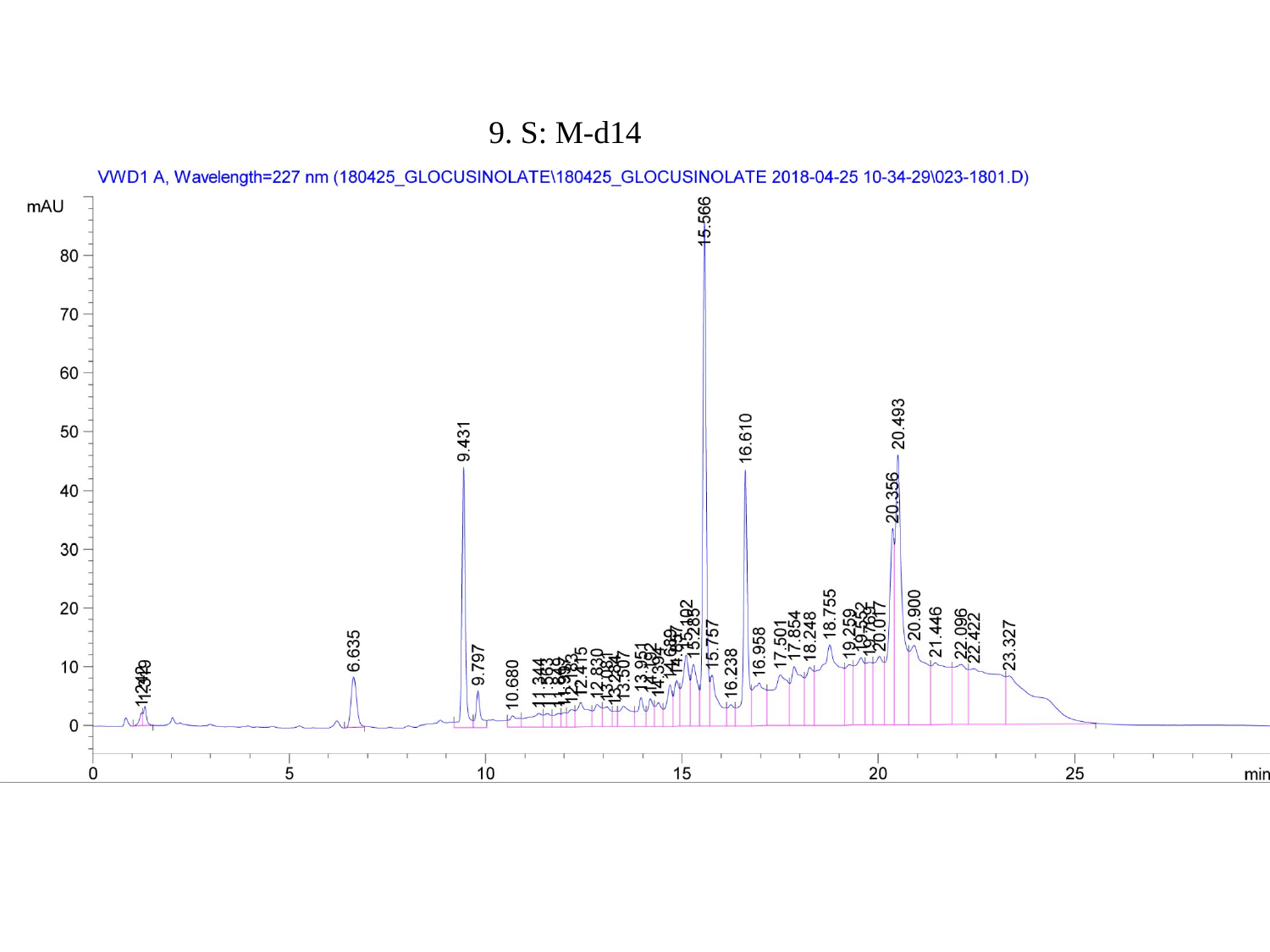

9. S: M-d14

## Slide 10
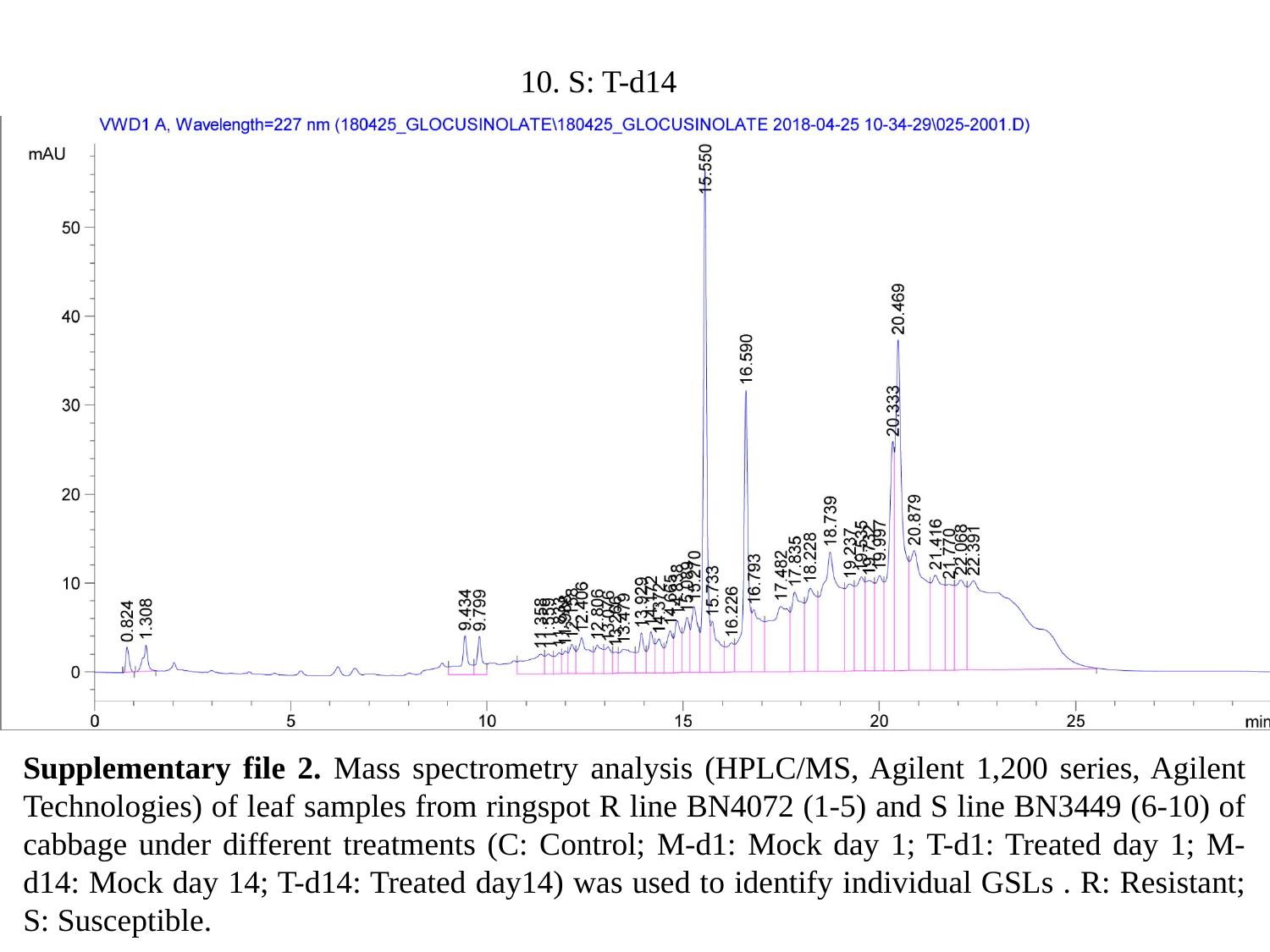

10. S: T-d14
Supplementary file 2. Mass spectrometry analysis (HPLC/MS, Agilent 1,200 series, Agilent Technologies) of leaf samples from ringspot R line BN4072 (1-5) and S line BN3449 (6-10) of cabbage under different treatments (C: Control; M-d1: Mock day 1; T-d1: Treated day 1; M-d14: Mock day 14; T-d14: Treated day14) was used to identify individual GSLs . R: Resistant; S: Susceptible.
