# Supplementary material for: Altered Glucosinolate Profiles and Expression of Glucosinolate Biosynthesis Genes in Ringspot-Resistant and Susceptible Cabbage Lines
Source: Int J Mol Sci. 2018 Sep 19;19(9):2833. doi: 10.3390/ijms19092833 (PMC6163659; doi:10.3390/ijms19092833)
Supplement: Supplementary file 1 [file ijms-19-02833-s001.zip › Supplementary tables.docx]

**Table S1.** Primer sequences [49-50] for the 38 glucosinolate biosynthesis related genes used in the relative expression analysis via qPCR in Ringspot R line BN4072 and S line BN3449 of cabbage.

| **Genes** | **cDNA  Size (bp)** | **Forward Primer Sequence** | **Reverse Primer Sequence** | **Product  Size (bp)** |
| --- | --- | --- | --- | --- |
| Transcription factor-related genes (11 genes) | | | | |
| *MYB28-Bol007795* | 558 | CCACACCAGTTCAGAGAGGT | GGGAAATGGATCGAAGTCAGC | 221 |
| *MYB28-Bol036286* | 615 | GAAGGTAGCTTGAATGCTAATAC | ATTCATGTAGTGCTCCTCATTC | 249 |
| *MYB28-Bol017019* | 426 | GTTGCGGCTAAGGTCACTTCT | CAGAAGTAGCGTTGATCTCATGC | 223 |
| *MYB28-Bol036743* | 426 | CTTGGGCGCTGCTACATTAC | ATCGTTCTCCTCGTTGTGGT | 241 |
| *MYB29-Bol008849* | 513 | CGCCCAAGACTTCTGAGTT | TGATATTGCCCATGGAAGCTG | 234 |
| *MYB34-Bol007760* | 843 | TG‍AAGGAGGATGGCGTACTC | CAGTTCGTCCCGCCAAATTA | 203 |
| *MYB34-Bol017062* | 951 | AAGGTGGATGGCGTACTCTC | TGTGAGTGGTTGGATCGACA | 279 |
| *MYB34-Bol036262* | 294 | CCCCGAGTTCTTTAGCAACC | TCCAAGTCCAGATCGTCTTCT | 198 |
| *MYB51-Bol013207* | 1002 | CCAGAGATTCCAGAGAAGC | CAAGTCACACTGCTACTACTAC | 233 |
| *MYB51-Bol030761* | 990 | CAGACAACTATATCGAGTAACG | TATCATTAACGGTCATCTGG | 274 |
| *MYB122-Bol026204* | 981 | GACCATTCCGAGACATTGCC | GCATCGTGGATCATGTGGAG | 284 |
| Aliphatic biosynthesis-related genes (10 genes) | | | | |
| *ST5b-Bol026201* | 1035 | CCGAGCCGTCAGAATTCAAG | GCTATGGCGAAAGTGAGAGC | 247 |
| *ST5b-Bol026202* | 1035 | AAGCCTTGACTTTCGCCATC | ACTTCACAACTGAGTCCGGT | 204 |
| *ST5c-Bol030757* | 1014 | CCACGCCCAAAACTTCTTCA | TGAGTGGAGAAGAGCGTGTT | 246 |
| *FMOGS-OX2-Bol010993* | 1386 | GAGAAGGTATCCGAGCCACA | GTCCACTGCAAACAACGACT | 200 |
| *FMOGS-OX5-Bol029100* | 1347 | CTTGCTCCAACGCTTTCCTT | CCTCAGCTCTCCAGTGTTCA | 280 |
| *FMOGS-OX5-Bol031350* | 1380 | GACACTACACAGAGCCTCGT | CCCCGGGAAGCTTCTCATAT | 234 |
| *AOP2-Bo2g102190* | 1104 | GGAACGTGTCTCCAAAACCC | TAGCACCATCACCAGCATCA | 354 |
| *AOP2-Bo3g052110* | 948 | CCAGGAAGTGAGAAGTGGGT | ACCAACATCCGCACCAGTAT | 552 |
| *AOP2-Bo9g006240* | 1032 | CCAGGAAGTGAGAAGTGGGT | TAGCACCATCACCAGCATCA | 517 |
| *GSL-OH-Bol033373* | 243 | GATTGTGCAAAAGGCTTGT | AGAGCATTAGGATTAGGAGGA | 188 |
| Indolic biosynthesis-related genes (17 genes) | | | | |
| *ST5a-Bol026200* | 1017 | GTCCGGTTGCAAGATGGTTT | CCTCTCCGGGTTCTCTTTGT | 214 |
| *ST5a-Bol039395* | 1014 | TGCCGTTTGTGAAGAGGTTG | CCCAATCTCCAACCTTCCCT | 210 |
| *CYP81F4-Bol032712* | 1506 | CGGTGGAGGAGAAGGAGAAA | CTGACACATGGCTCGTAACG | 226 |
| *CYP81F4-Bol032714* | 960 | ACCCTGGTGAATACTTGCCA | GAAACACACTGAAGCAGAAC | 239 |
| *CYP81F4-Bol028918* | 1503 | GTTTGCGGCATCAGAGACAT | GAATAGTCCACGCGTTCACC | 299 |
| *CYP81F1-Bol017375* | 369 | AAGCAGAGCGGTTCAAGAAG | GCGTGACCATTGTGTTACCA | 204 |
| *CYP81F1-Bol017376* | 246 | CCGTCTCCTTCAACGGTTCT | CGACGTATTTACCGGTGAGC | 170 |
| *CYP81F1-Bol028913* | 1500 | GAGACCTCCGCAGTAACCTT | GTCCTCCGTCGGTCTTCTAG | 222 |
| *CYP81F1-Bol028914* | 1497 | CTTTCCAACTGACGGCCAAA | CGTTAGGTCCGAGAAAAGCG | 257 |
| *CYP81F2-Bol012237* | 933 | GCAGCCGTGACACTAGAATG | TCCGCCAATCTTGAGGTCTT | 231 |
| *CYP81F2-Bol014239* | 1482 | TTGTACCGCGTTCTCCTTCT | GACACCATCCTCTGACCCAA | 238 |
| *CYP81F2-Bol026044* | 1482 | TTCTCCCTACGTTACGGCTC | CTACGAACGGAGAGGAGTCC | 251 |
| *CYP81F3-Bol028919* | 1500 | TAACAGCGGAGGAGAAGACG | CACCTTCTAACTGGGCCTGA | 260 |
| *CYP81F3-Bol032711* | 1492 | CCGTCTCACCAACTTCCTCT | CTTCTCAAAGCTCCCTCCCA | 292 |
| *IGMT1-Bol007029* | 1119 | GTGTTCCTCTCACCTTCCGA | GTGTTGAGGAAGACGCTGTC | 260 |
| *IGMT1-Bol020663* | 342 | AGATGCCATGATCTTGAAACGT | CCAGCAATGATAAGCCTGACA | 298 |
| *IGMT2-Bol007030* | 1125 | AGCCTTTCCCATGGTTCTCA | TCTCTCGCCCTTTCCAAACT | 223 |

**Table S2.** Heat maps comparing Ringspot R line BN4072 and S line BN3449 of cabbage shows fold changes in expression of transcription factor related genes in *Mycosphaerella brassicicola*-inoculated leaf samples compared to respective mock-treated samples. d1: day 1; d3: day 3; d7: day 7; d14: day 14; R: Resistant; S: Susceptible.

| Genes | Treatments | BN4072 | BN3449 |
| --- | --- | --- | --- |
| *MYB28-Bol007795* | d1 | 0.94 | 1.7 |
|  | d3 | 0.75 | 2.20 |
|  | d7 | 1.14 | 3.57 |
|  | d14 | 2.28 | 3.59 |
| *MYB28-Bol036286* | d1 | 0.75 | 3.8 |
|  | d3 | 1.82 | 1.89 |
|  | d7 | 2.45 | 2.65 |
|  | d14 | 11.52 | 2.65 |
| *MYB28-Bol017019* | d1 | 1.23 | 6.1 |
|  | d3 | 1.15 | 3.89 |
|  | d7 | 0.87 | 0.74 |
|  | d14 | 0.56 | 0.57 |
| *MYB28-Bol036743* | d1 | 0.85 | 4.0 |
|  | d3 | 0.81 | 1.26 |
|  | d7 | 1.97 | 1.54 |
|  | d14 | 4.57 | 0.73 |
| *MYB29-Bol008849* | d1 | 1.31 | 11.0 |
|  | d3 | 0.90 | 3.26 |
|  | d7 | 0.40 | 1.46 |
|  | d14 | 3.15 | 1.03 |
| *MYB34-Bol017062* | d1 | 0.88 | 0.5 |
|  | d3 | 0.88 | 1.47 |
|  | d7 | 1.11 | 1.45 |
|  | d14 | 9.25 | 1.16 |
| *MYB34-Bol007760* | d1 | 2.55 | 1.0 |
|  | d3 | 2.59 | 0.43 |
|  | d7 | 0.88 | 0.73 |
|  | d14 | 0.89 | 0.87 |
| *MYB34-Bol036262* | d1 | 2.40 | 8.4 |
|  | d3 | 1.15 | 1.79 |
|  | d7 | 1.30 | 1.99 |
|  | d14 | 4.46 | 0.99 |
| *MYB51-Bol013207* | d1 | 2.00 | 0.9 |
|  | d3 | 1.49 | 0.36 |
|  | d7 | 1.90 | 0.62 |
|  | d14 | 1.69 | 0.74 |
| *MYB51-Bol030761* | d1 | 13.13 | 4.9 |
|  | d3 | 0.99 | 2.52 |
|  | d7 | 1.32 | 1.42 |
|  | d14 | 0.76 | 2.07 |
| *MYB122-Bol026204* | d1 | 1.45 | 8.0 |
|  | d3 | 0.62 | 2.26 |
|  | d7 | 0.76 | 1.97 |
|  | d14 | 1.39 | 0.63 |

**Table S3.** Heat maps comparing Ringspot R line BN4072 and S line BN3449 of cabbage shows fold changes in expression of aliphatic glucosinolate biosynthesis genes in *Mycosphaerella brassicicola*-inoculated leaf samples compared to respective mock-treated samples. d1: day 1; d3: day 3; d7: day 7; d14: day 14; R: Resistant; S: Susceptible.

| Genes | Treatments | BN4072 | BN3449 |
| --- | --- | --- | --- |
| *ST5b-Bol026202* | d1 | 8.60 | 4.1 |
|  | d3 | 5.64 | 5.48 |
|  | d7 | 1.31 | 0.64 |
|  | d14 | 12.07 | 0.99 |
| *ST5b-Bol026201* | d1 | 6.37 | 0.9 |
|  | d3 | 1.47 | 0.85 |
|  | d7 | 0.86 | 0.83 |
|  | d14 | 1.30 | 0.85 |
| *ST5c-Bol030757* | d1 | 4.42 | 2.4 |
|  | d3 | 3.15 | 1.96 |
|  | d7 | 1.15 | 2.22 |
|  | d14 | 27.31 | 0.41 |
| *FMOGS-OX2-Bol010993* | d1 | 0.97 | 2.6 |
|  | d3 | 1.19 | 0.46 |
|  | d7 | 1.39 | 0.52 |
|  | d14 | 1.72 | 0.83 |
| *FMOGS-OX5-Bol029100* | d1 | 1.27 | 1.1 |
|  | d3 | 2.83 | 0.95 |
|  | d7 | 1.69 | 1.22 |
|  | d14 | 0.84 | 1.00 |
| *FMOGS-OX5-Bol031350* | d1 | 0.84 | 0.4 |
|  | d3 | 0.75 | 0.57 |
|  | d7 | 1.17 | 1.10 |
|  | d14 | 0.94 | 1.19 |
| *GSL-OH-Bol033373* | d1 | 3.05 | 1.9 |
|  | d3 | 1.76 | 2.43 |
|  | d7 | 0.56 | 1.08 |
|  | d14 | 4.43 | 1.04 |
| *AOP2-Bo2g102190* | d1 | 0.93 | 8.3 |
|  | d3 | 0.33 | 3.19 |
|  | d7 | 0.21 | 2.62 |
|  | d14 | 0.75 | 1.26 |
| *AOP2-Bo3g052110* | d1 | 1.47 | 6.3 |
|  | d3 | 0.49 | 14.05 |
|  | d7 | 0.68 | 1.59 |
|  | d14 | 0.84 | 1.71 |
| *AOP2-Bo9g006240* | d1 | 1.14 | 5.4 |
|  | d3 | 1.53 | 6.21 |
|  | d7 | 0.96 | 1.64 |
|  | d14 | 1.23 | 2.79 |

**Table S4.** Heat maps comparing Ringspot R line BN4072 and S line BN3449 of cabbage shows fold changes in expression of indolic glucosinolate-biosynthesis related genes in *Mycosphaerella brassicicola*-inoculated leaf samples compared to respective mock-treated samples. d1: day 1; d3: day 3; d7: day 7; d14: day 14; R: Resistant; S: Susceptible.

| Genes | Treatments | BN4072 | BN3449 |
| --- | --- | --- | --- |
| *ST5a-Bol039395* | d1 | 0.82 | 3.1 |
|  | d3 | 0.31 | 1.17 |
|  | d7 | 0.71 | 3.19 |
|  | d14 | 4.68 | 0.66 |
| *ST5a-Bol026200* | d1 | 2.56 | 4.5 |
|  | d3 | 0.94 | 2.01 |
|  | d7 | 1.53 | 0.43 |
|  | d14 | 4.60 | 0.49 |
| *CYP81F1-Bol028913* | d1 | 0.55 | 3.9 |
|  | d3 | 0.45 | 2.77 |
|  | d7 | 1.03 | 1.26 |
|  | d14 | 5.60 | 1.16 |
| *CYP81F1-Bol028914* | d1 | 1.02 | 6.2 |
|  | d3 | 0.57 | 30.43 |
|  | d7 | 1.33 | 1.55 |
|  | d14 | 0.52 | 1.42 |
| *CYP81F1-Bol017375* | d1 | 0.90 | 1.7 |
|  | d3 | 0.78 | 1.30 |
|  | d7 | 1.01 | 1.38 |
|  | d14 | 0.56 | 0.59 |
| *CYP81F1-Bol017376* | d1 | 0.41 | 6.4 |
|  | d3 | 0.25 | 2.44 |
|  | d7 | 0.32 | 2.24 |
|  | d14 | 0.33 | 0.65 |
| *CYP81F2-Bol012237* | d1 | 1.52 | 0.6 |
|  | d3 | 0.63 | 0.77 |
|  | d7 | 1.02 | 1.38 |
|  | d14 | 0.97 | 1.28 |
| *CYP81F2-Bol014239* | d1 | 2.17 | 1.8 |
|  | d3 | 0.35 | 0.62 |
|  | d7 | 0.77 | 1.23 |
|  | d14 | 1.13 | 0.26 |
| *CYP81F2-Bol026044* | d1 | 12.86 | 3.3 |
|  | d3 | 2.25 | 1.58 |
|  | d7 | 0.71 | 1.10 |
|  | d14 | 16.08 | 1.25 |
| *CYP81F3-Bol032711* | d1 | 2.34 | 8.0 |
|  | d3 | 9.26 | 5.69 |
|  | d7 | 0.31 | 1.25 |
|  | d14 | 0.55 | 0.57 |
| *CYP81F3-Bol028919* | d1 | 1.11 | 4.3 |
|  | d3 | 0.42 | 0.54 |
|  | d7 | 0.38 | 3.43 |
|  | d14 | 0.64 | 2.97 |
| *CYP81F4-Bol032712* | d1 | 15.44 | 6.1 |
|  | d3 | 1.59 | 2.05 |
|  | d7 | 1.27 | 1.06 |
|  | d14 | 1.34 | 0.76 |
| *CYP81F4-Bol032714* | d1 | 15.35 | 22.8 |
|  | d3 | 3.02 | 7.02 |
|  | d7 | 0.95 | 1.20 |
|  | d14 | 0.80 | 1.02 |
| *CYP81F4-Bol028918* | d1 | 1.80 | 2.5 |
|  | d3 | 0.88 | 5.61 |
|  | d7 | 0.32 | 1.62 |
|  | d14 | 0.59 | 3.39 |
| *IGMT1-Bol007029* | d1 | 0.93 | 2.0 |
|  | d3 | 0.91 | 1.12 |
|  | d7 | 3.05 | 0.99 |
|  | d14 | 5.01 | 0.83 |
| *IGMT1-Bol020663* | d1 | 1.17 | 11.4 |
|  | d3 | 0.73 | 0.65 |
|  | d7 | 4.20 | 1.77 |
|  | d14 | 6.53 | 1.60 |
| *IGMT2-Bol007030* | d1 | 0.35 | 4.3 |
|  | d3 | 0.31 | 0.88 |
|  | d7 | 1.44 | 0.92 |
|  | d14 | 1.03 | 1.18 |

**Table S5.** Heat maps comparing Ringspot R line BN4072 and S line BN3449 of cabbage shows fold changes in idividual glucosinolate component in *Mycosphaerella brassicicola*-inoculated leaf samples compared to respective mock-treated samples. d1: day 1; d14: day 14; R: Resistant; S: Susceptible.

| Glucosinolate component | Treatments | BN4072 | BN3449 |
| --- | --- | --- | --- |
| Glucoiberin (GIB) | d1 | 0.55 | 1.62 |
|  | d14 | 0.87 | 0.01 |
| Sinigrin (SIN) | d1 | 0.92 | 1.03 |
|  | d14 | 0.58 | 0.21 |
| Gluconapin (GNA) | d1 | 1.43 | 0.82 |
|  | d14 | 2.63 | 0.95 |
| Glucoiberverin (GIV) | d1 | 3.06 | 1.15 |
|  | d14 | 8.58 | 1.04 |
| Glucoerucin (GER) | d1 | 0.72 | 1.33 |
|  | d14 | 1.28 | 1.12 |
| Hydroxyglucobrassicin (HGBS) | d1 | 1.18 | 1.17 |
|  | d14 | 2.17 | 1.00 |
| Glucobrassicin (GBS) | d1 | 0.24 | 1.73 |
|  | d14 | 3.82 | 0.93 |
| Methoxyglucobrassicin (MGBS) | d1 | 2.01 | 1.36 |
|  | d14 | 6.20 | 0.85 |
| Neoglucobrassicin (NGBS) | d1 | 0.84 | 1.09 |
|  | d14 | 1.93 | 0.98 |

**Table S6.** Test statistic and p-values for expression of glucosinolate biosynthesis genes in Ringspot R line BN4072 and S line BN3449 of cabbage with 1 and treatment interactions with 17 degrees of freedom, respectively. R: Resistant; S: Susceptible.

| Relative expression of genes | R & S lines | | R & S lines and treatment Interactions | |
| --- | --- | --- | --- | --- |
|  | F value | P value | F value | P value |
| *MYB28-Bol007795* | 12.88 | <0.01 | 25.22 | <0.01 |
| *MYB28-Bol036286* | 20.49 | <0.01 | 61.89 | <0.01 |
| *MYB28-Bol017019* | 0.25 | 0.620 | 118.79 | <0.01 |
| *MYB28-Bol036743* | 8.83 | 0.005 | 54.24 | <0.01 |
| *MYB29-Bol008849* | 8.36 | 0.007 | 35.15 | <0.01 |
| *MYB34-Bol007760* | 4.89 | 0.034 | 331.06 | <0.01 |
| *MYB34-Bol017062* | 11.68 | 0.002 | 113.20 | <0.01 |
| *MYB34-Bol036262* | 2.09 | 0.157 | 59.83 | <0.01 |
| *MYB51-Bol013207* | 5.04 | 0.031 | 32.18 | <0.01 |
| *MYB51-Bol030761* | 3.93 | 0.055 | 53.21 | <0.01 |
| *MYB122-Bol026204* | 4.22 | 0.048 | 157.42 | <0.01 |
| *ST5a-Bol026200* | 0.60 | 0.445 | 36.10 | <0.01 |
| *ST5a-Bol039395* | 8.22 | 0.007 | 37.23 | <0.01 |
| *ST5b-Bol026202* | 2.34 | 0.135 | 16.05 | <0.01 |
| *ST5b-Bol026201* | 62.54 | <0.01 | 53.86 | <0.01 |
| *ST5c-Bol030757* | 12.24 | 0.001 | 89.54 | <0.01 |
| *FMOGS-OX2-Bol010993* | 11.57 | 0.002 | 48.93 | <0.01 |
| *FMOGS-OX5-Bol029100* | 16.31 | <0.01 | 83.79 | <0.01 |
| *FMOGS-OX5-Bol031350* | 32.27 | <0.01 | 84.89 | <0.01 |
| *AOP2-Bo2g102190* | 4.47 | 0.042 | 201.92 | <0.01 |
| *AOP2-Bo3g052110* | 5.88 | 0.021 | 85.91 | <0.01 |
| *AOP2-Bo9g006240* | 8.58 | 0.006 | 33.40 | <0.01 |
| *GSL-OH-Bol033373* | 9.85 | 0.004 | 184.19 | <0.01 |
| *CYP81F4-Bol032712* | 0.82 | 0.371 | 125.00 | <0.01 |
| *CYP81F4-Bol032714* | 0.03 | 0.873 | 57.39 | <0.01 |
| *CYP81F4-Bol028918* | 2.56 | 0.119 | 36.64 | <0.01 |
| *CYP81F1-Bol017375* | 11.19 | 0.002 | 15.64 | <0.01 |
| *CYP81F1-Bol017376* | 3.97 | 0.054 | 47.04 | <0.01 |
| *CYP81F1-Bol028913* | 5.75 | 0.022 | 36.12 | <0.01 |
| *CYP81F1-Bol028914* | 0.03 | 0.859 | 163.05 | <0.01 |
| *CYP81F2-Bol012237* | 163.25 | <0.01 | 34.25 | <0.01 |
| *CYP81F2-Bol014239* | 25.64 | <0.01 | 35.53 | <0.01 |
| *CYP81F2-Bol026044* | 6.18 | 0.018 | 34.07 | <0.01 |
| *CYP81F3-Bol028919* | 7.89 | 0.008 | 42.31 | <0.01 |
| *CYP81F3-Bol032711* | 1.12 | 0.298 | 27.39 | <0.01 |
| *IGMT1-Bol007029* | 0.32 | 0.578 | 83.92 | <0.01 |
| *IGMT1-Bol020663* | 0.69 | 0.413 | 154.12 | <0.01 |
| *IGMT2-Bol007030* | 2.40 | 0.131 | 39.88 | <0.01 |

**Table S7.** Test statistic and p-values for glucosinolate component in Ringspot R line BN4072 and S line BN3449 of cabbage with 1 and treatment interactions with 9 degrees of freedom, respectively. R: Resistant; S: Susceptible.

| Glucosinolate component | R & S lines | | R & S lines and treatment interaction | |
| --- | --- | --- | --- | --- |
|  | F value | P value | F value | P value |
| Glucoiberin (GIB) | 7.25 | 0.015 | 35.92 | <0.01 |
| Sinigrin (SIN) | 0.03 | 0.857 | 41.86 | <0.01 |
| Gluconapin (GNA) | 69.77 | <0.01 | 14.22 | <0.01 |
| Glucoiberverin (GIV) | 4.58 | 0.046 | 15.05 | <0.01 |
| Glucoerucin (GER) | 39.87 | <0.01 | 3.83 | 0.024 |
| Hydroxyglucobrassicin (HGBS) | 11.39 | 0.003 | 4.43 | 0.015 |
| Glucobrassicin (GBS) | 6.15 | 0.023 | 11.27 | <0.01 |
| Methoxyglucobrassicin (MGBS) | 3.15 | 0.093 | 6.46 | 0.004 |
| Neoglucobrassicin (NGBS) | 16.30 | 0.001 | 9.22 | 0.001 |

**Table S8.** Component loadings of cabbage Ringspot R line BN4072 and S line BN3449, Glucosinolate components and Glucosinolate biosynthesis pathway gene responses as determined by the principle component analysis (PCA). R: Resistant; S: Susceptible.

| Variable | PC1 | PC2 | PC3 | PC4 |
| --- | --- | --- | --- | --- |
| **Glucosinolate biosynthesis pathway genes** | | | | |
| MYB28-Bol007795 | 0.162 | 0.036 | -0.026 | -0.007 |
| MYB28-Bol036286 | 0.001 | -0.234 | -0.045 | 0.073 |
| MYB28-Bol017019 | 0.176 | -0.100 | 0.008 | 0.115 |
| MYB28-Bol036743 | 0.194 | 0.004 | -0.085 | 0.098 |
| MYB29-Bol008849 | 0.182 | 0.013 | -0.157 | 0.114 |
| MYB34-Bol017062 | -0.075 | -0.084 | -0.242 | 0.055 |
| MYB34-Bol007760 | 0.162 | 0.042 | 0.163 | -0.157 |
| MYB34-Bol036262 | 0.021 | -0.139 | -0.287 | -0.016 |
| MYB51-Bol013207 | 0.112 | 0.138 | 0.113 | -0.091 |
| MYB51-Bol030761 | 0.189 | -0.043 | -0.092 | -0.112 |
| MYB122-Bol026204 | 0.195 | -0.016 | -0.081 | 0.099 |
| ST5a-Bol039395 | 0.197 | 0.024 | 0.02 | 0.054 |
| ST5a-Bol026200 | 0.186 | -0.090 | -0.017 | -0.100 |
| ST5b-Bol026202 | -0.03 | -0.158 | -0.192 | 0.337 |
| ST5b-Bol026201 | 0.137 | 0.125 | 0.095 | -0.273 |
| ST5c-Bol030757 | 0.020 | -0.211 | -0.111 | -0.296 |
| FMOGS-OX2-Bol010993 | 0.203 | 0.024 | 0.006 | 0.04 |
| FMOGS-OX5-Bol029100 | 0.156 | 0.107 | 0.168 | -0.058 |
| FMOGS-OX5-Bol031350 | 0.034 | 0.210 | 0.22 | -0.148 |
| GSL-OH-Bol033373 | 0.198 | 0.019 | -0.001 | -0.014 |
| CYP81F1-Bol028913 | 0.195 | -0.018 | -0.043 | 0.128 |
| CYP81F1-Bol028914 | 0.052 | -0.244 | 0.048 | -0.039 |
| CYP81F1-Bol017375 | 0.109 | -0.225 | 0.124 | 0.082 |
| CYP81F1-Bol017376 | 0.115 | -0.171 | 0.037 | 0.273 |
| CYP81F2-Bol012237 | 0.092 | 0.218 | 0.149 | -0.141 |
| CYP81F2-Bol014239 | 0.185 | 0.104 | 0.044 | 0.020 |
| CYP81F2-Bol026044 | 0.090 | -0.133 | -0.121 | -0.348 |
| CYP81F3-Bol032711 | 0.190 | -0.080 | -0.078 | -0.032 |
| CYP81F3-Bol028919 | 0.199 | 0.002 | -0.055 | 0.085 |
| CYP81F4-Bol032712 | 0.089 | -0.159 | -0.076 | -0.326 |
| CYP81F4-Bol032714 | 0.087 | -0.177 | -0.08 | -0.299 |
| CYP81F4-Bol028918 | 0.184 | -0.094 | -0.028 | -0.018 |
| IGMT1-Bol007029 | 0.187 | -0.074 | 0.065 | 0.065 |
| IGMT1-Bol020663 | -0.191 | 0.032 | -0.112 | 0.096 |
| IGMT2-Bol007030 | 0.196 | 0.001 | 0.002 | 0.131 |
| AOP2-Bo2g102190 | 0.193 | -0.023 | -0.083 | 0.115 |
| AOP2-Bo3g052110 | 0.198 | -0.015 | -0.077 | 0.066 |
| AOP2-Bo9g006240 | 0.184 | 0.026 | -0.109 | 0.082 |
| **Glucosinolate components** | | | | |
| Glucoiberin | 0.184 | 0.053 | 0.115 | 0.044 |
| Sinigrin | 0.121 | -0.108 | 0.257 | -0.053 |
| Gluconapin | 0.033 | 0.267 | 0.003 | -0.090 |
| Glucoiberverin | -0.013 | -0.171 | -0.277 | -0.081 |
| 4-hydroxyglucobrassicin | 0.010 | -0.22 | -0.222 | -0.036 |
| Glucoerucin | 0.100 | 0.249 | -0.055 | 0.0360 |
| Glucobrassicin | -0.110 | -0.072 | -0.160 | 0.227 |
| 4-Methoxyglucobrassicin | -0.039 | 0.187 | -0.296 | -0.038 |
| Neoglucobrassicin | 0.068 | 0.202 | -0.190 | 0.051 |
| % Variation explained | 47.7 | 23.0 | 11.0 | 8.5 |
| **Cabbage lines** | **Mean PC scores (±SD)** | | | |
| R-line BN4072 | -2.15 ± 1.989 | -2.749 ± 1.960 b | -0.16 ± 2.51 | 0.27 ± 2.84 |
| S-line BN3449 | 2.15 ± 6.18 | 2.749 ± 1.773 a | 0.16 ± 2.45 | -0.267 ± 1.170 |
| P-value | 0.177 | 0.002 | 0.844 | 0.707 |
